# Disruption of the productive encounter complex results in dysregulation of DIAPH1 activity

**DOI:** 10.1016/j.jbc.2023.105342

**Published:** 2023-10-12

**Authors:** Gregory G. Theophall, Lisa M.S. Ramirez, Aaron Premo, Sergey Reverdatto, Michaele B. Manigrasso, Gautham Yepuri, David S. Burz, Ravichandran Ramasamy, Ann Marie Schmidt, Alexander Shekhtman

**Affiliations:** 1 Department of Chemistry, State University of New York at Albany, Albany, New York, USA; 2 Department of Medicine, Diabetes Research Program, New York University Grossman School of Medicine, New York, New York, USA

**Keywords:** actin polymerization, diabetes, intrinsically disordered regions, protein–protein interactions, NMR, mass spectrometry, MS, cross-linking, encounter complex, DRF, receptor for advanced glycation end products, mDia1, DIAPH1

## Abstract

The diaphanous-related formin, Diaphanous 1 (DIAPH1), is required for the assembly of Filamentous (F)-actin structures. DIAPH1 is an intracellular effector of the receptor for advanced glycation end products (RAGE) and contributes to RAGE signaling and effects such as increased cell migration upon RAGE stimulation. Mutations in *DIAPH1*, including those in the basic “RRKR” motif of its autoregulatory domain, diaphanous autoinhibitory domain (DAD), are implicated in hearing loss, macrothrombocytopenia, and cardiovascular diseases. The solution structure of the complex between the N-terminal inhibitory domain, DID, and the C-terminal DAD, resolved by NMR spectroscopy shows only transient interactions between DID and the basic motif of DAD, resembling those found in encounter complexes. Cross-linking studies placed the RRKR motif into the negatively charged cavity of DID. Neutralizing the cavity resulted in a 5-fold decrease in the binding affinity and 4-fold decrease in the association rate constant of DAD for DID, indicating that the RRKR interactions with DID form a productive encounter complex. A DIAPH1 mutant containing a neutralized RRKR binding cavity shows excessive colocalization with actin and is unresponsive to RAGE stimulation. This is the first demonstration of a specific alteration of the surfaces responsible for productive encounter complexation with implications for human pathology.

Actin polymerization is a central cellular process that is responsible for cell migration, cytokinesis, and cell proliferation (1, 2). A diaphanous-related formin (DRF), Diaphanous 1 or DIAPH1, is a dimeric, multidomain, multifunctional DRF that possesses actin polymerase activity, promoting the synthesis of linear cables of actin in response to various stimuli (3, 4, 5, 6), such as those generated by the signaling of the receptor for advanced glycation end products (RAGE) (7, 8, 9, 10). The N-terminal region of DIAPH1 has a GTPase-binding domain, followed by the diaphanous inhibitory domain (DID), that contains an armadillo repeat region (ARR), and an interdomain helix (IH, also called α5B), central dimerization (DD), and coiled-coil domains. The C-terminal region of DIAPH1 has the formin homology 1 and 2 domains (FH1 and FH2), which are involved in actin polymerization (3, 4), a T-helix, and the diaphanous autoinhibitory domain (DAD), that binds intramolecularly to DID to maintain DIAPH1 in the autoinhibited state (Fig. 1*A*) (11, 12, 13).

**Figure 1:**
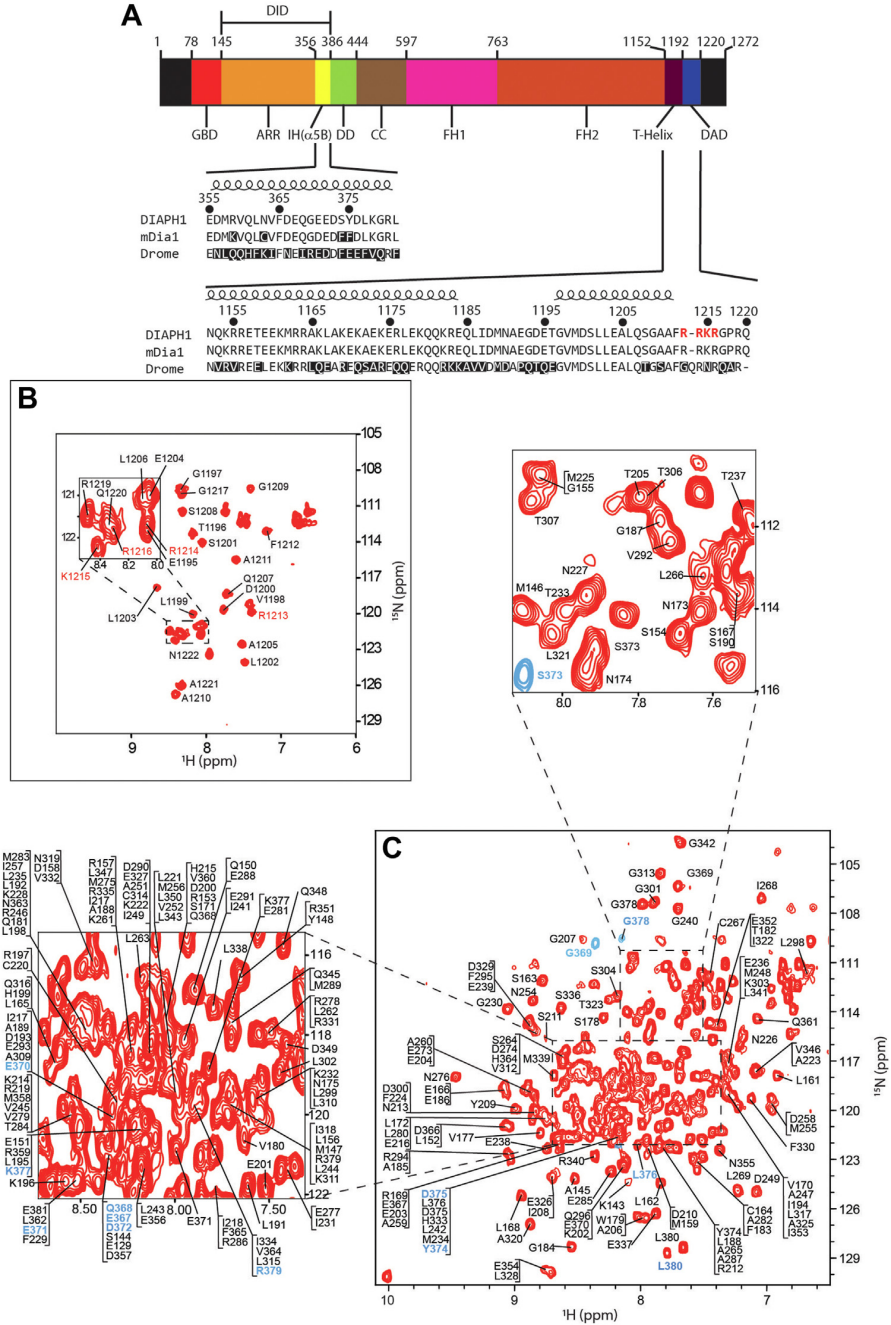
**DID and DAD**^**M1199L**^**domains of DIAPH1 form a complex.***A*, schematic representation of the domain structure of DIAPH1. Below are amino acid sequences of the IH and C-terminal region from different organisms: DIAPH1, human (UniProt entry O60610), mouse DIAPH1, mouse (UniProt entry O08808), Drome, fruit fly (UniProt entry P48608). Numbering system corresponds to DIAPH1. Residues that are different from DIAPH1 are highlighted in *black, helical regions* are indicated by the coiled ribbon above the sequences and RRKR motif is in *red*. *B*, ^1^H-^15^N HSQC spectrum of [*U*-^15^N]-DAD^M1199L^ bound to DID shows well-dispersed backbone amide proton and nitrogen resonances, indicating that DAD^M1199L^ acquires structure upon binding to DID (Fig. S1*A*). Peaks of the RRKR motif residues are labeled in *red*. *C*, ^1^H-^15^N HSQC spectrum of [*U*-^15^N]-DID bound to DAD^M1199L^ indicates that the complex is structured. Peaks labeled in *blue* correspond to conformer B. *Blue peaks* were used in thermodynamic calculations. DAD, diaphanous autoinhibitory domain; DIAPH1, Diaphanous 1; DID, diaphanous inhibitory domain; HSQC, heteronuclear single–quantum coherence.

Although no structural information is available for human DIAPH1, structures of the murine homolog of DIAPH1, with 90.3% sequence identity to human DIAPH1 (14), provide a guide to understanding the disparate functions and regulation of DIAPH1. Crystal structures of complexes between the N- and C-terminal fragments of mouse DIAPH1, also called mDia1, present a glimpse into the autoinhibited state (11, 15, 16, 17). In DAD, the segment directly N terminal to the RRKR motif forms a helix and a turn, anchored at F1195, that binds to DID to maintain mouse DIAPH1 in an autoinhibited state. The RRKR motif, which is also found in many unrelated proteins, such as coronavirus spike protein (18), proplatelet-derived growth factor A (19), chloroplast signal recognition particle 54 (20), and nuclear valosin–containing protein-like 2 (21), is likely flexible because it is either absent (11, 15) or poorly resolved (16, 17) but is oriented proximal to negatively charged patches on DID (22, 23) and contributes to the binding (11). It is not clear why mutations of this flexible region of DAD result in DIAPH1 activation (22, 24). In addition, the T-helix in mouse DIAPH1 blocks the negatively charged patches on DID, thereby precluding transient interactions between DID and the RRKR motif of DAD (15, 17). Crystal structures of the N- and C-terminal fragments of mouse DIAPH1 display different angles between DID and DD in mouse DIAPH1 dimers, suggesting flexibility in the IH that links DID and DD (11, 15, 23, 25).

DIAPH1 is widely expressed in various cell types, especially muscle (26) and kidney cells (27). Mice devoid of *Diaph1* are viable (27, 28). In humans, loss-of-function mutations in *DIAPH1* are associated with immunodeficiency, microcephaly, and mitochondrial dysfunction (29, 30); whereas mutations in DAD, resulting from deletion or modification of the RRKR motif (22, 31), for example, in the DIAPH1-(R1213X) where the RRKR motif is deleted, confer gain-of-function that have been implicated in autosomal dominant hearing loss (24) and macrothrombocytopenia (32).

To understand the structural determinants of DIAPH1 regulation, DID–DAD interactions were characterized by using solution NMR spectroscopy, cross-linking with mass spectrometry (MS), fluorescence spectroscopy, and functional assays. Flexible regions of DID were identified in the IH. The results also revealed that binding of RRKR to DID is transient and forms a productive encounter complex (33, 34) that does not compete with the T-helix. These observations allowed a consistent picture of DIAPH1 regulation to be proposed.

## Results

### DID–DAD^M1199L^ complex stoichiometry and affinity

**Figure 2:**
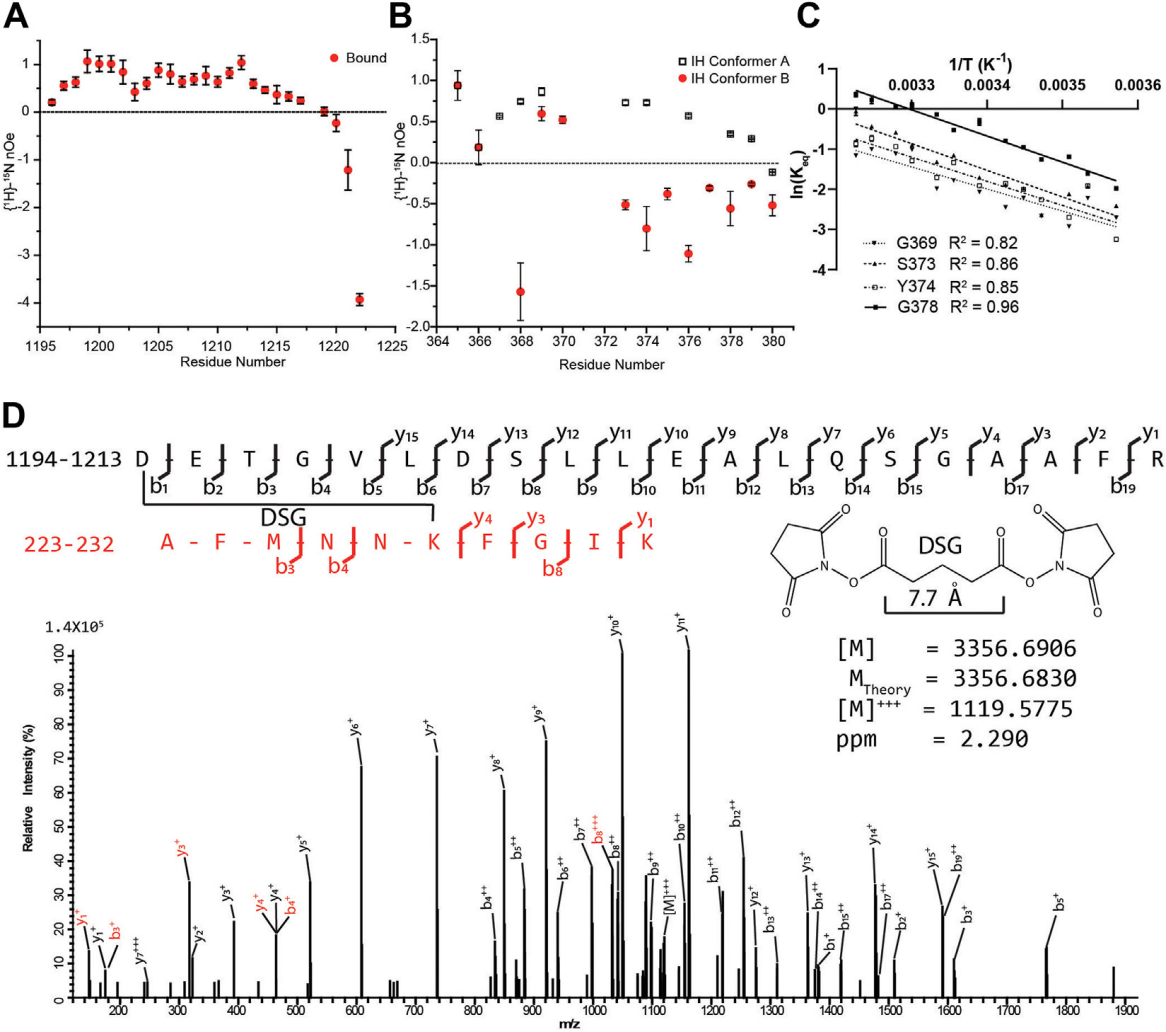
**DAD**^**M1199L**^**and interhelical domain of DIAPH1 are flexible.***A*, steady state {^1^H}-^15^N nOes of DAD^M1199L^bound to DID showed that the structural region of DAD^M1199L^ spans from G1197 to R1213 (Fig. S1*A*). *B*, steady state {^1^H}-^15^N nOe of the ^13^C-terminal DID residues of IH showed the presence of two conformers: a structured conformer A (*white squares*) and an unstructured conformer B (*red circles*). *C*, van’t Hoff analysis of selected DID C-terminal residues of IH showed an entropically driven equilibrium between conformers A and B. The temperature dependence of the equilibrium constant, K_eq_, resolved ΔH, ΔS, and ΔG for the unstructured to structured transition (Table S1). *D*, high-energy collision MS spectrum obtained for the DSG cross-linked product between K228 of DID and the N terminus of DAD^M1199L^ at 1119.036 *m/z*. For clarity, only the molecular ion, and the y and b peptidic fragmentations are labeled. The cross-linked peptides are shown in *black* and *red* along with the cross-linker DSG. The experimental mass of 3356.6906 Da is in good agreement with the theoretical mass of 3356.6830 Da calculated from putative elemental composition. Unassigned peaks are from water or ammonia loss, and/or nonpeptidic bond fragmentation. MS data was deposited to https://repository.jpostdb.org/ under acquisition codes JPST002156 and PXD042130. DAD, diaphanous autoinhibitory domain; DIAPH1, Diaphanous 1; DID, diaphanous inhibitory domain; DSG, disuccinimidyl glutarate.

The DID–DAD interaction was characterized by using a combination of solution NMR spectroscopy and cross-linking MS (Figs. 1 and 2). Residues 142 to 380 of DIAPH1, corresponding to DID, and residues 1194 to 1222, corresponding to DAD, were bacterially overexpressed for labeling and NMR spectroscopy (Fig. 1*A*). DAD was expressed as an insoluble fusion protein containing an N-terminal his-tag and modified tryptophan leader sequence (35, 36), and the resulting gene product accumulated in inclusion bodies. DAD methionine 1199 was conservatively (37) changed to a leucine, DAD^M1199L^, to ensure that only a single cyanogen bromide cleavage site was present so that cleavage of the fusion protein would yield soluble DAD^M1199L^ peptide. This substitution did not change the binding to DID (Figs. S1 and S2).

The stoichiometry of the interaction between DID and DAD^M1199L^ was determined by titrating 100 μM [*U*-^15^N]-DAD^M1199L^ with unlabeled DID (Figs. 1*B* and S1*A*). Chemical shifts (δ) and cross peak intensities (I) were monitored by using heteronuclear single–quantum coherence (^1^H-^15^N HSQC) NMR spectroscopy (38) (Fig. S1*A*). Changes in δ and I were observed at substoichiometric amounts of unlabeled DID. Negligible perturbations occurred beyond a 1:1 mole ratio, indicating a 1:1 stoichiometry for the complex. The strength of the DID–DAD^M1199L^ interaction was measured using tryptophan fluorescence spectroscopy and yielded a macroscopic binding constant of 270 ± 100 nM, in agreement with that reported for WT DAD (11) (Fig. S1*B*).

### DAD^M1199L^ peptide acquires structure when bound to DID

The ^1^H-^15^N HSQC, spectrum of [*U*-^15^N]-DAD^M1199L^, is shown in Figure 1*B*; 27 out of 29 residues were assigned. Changes in amide resonances of DAD^M1199L^ upon complex formation with DID suggest that free DAD^M1199L^ undergoes a structural transition when bound (Fig. S1*A*). Steady-state heteronuclear {^1^H}-^15^N nuclear Overhauser effect ({^1^H}-^15^N nOe) (38) was used to identify structured regions in bound DAD^M1199L^ (Fig. 2*A*). Negative {^1^H}-^15^N nOes indicate a high degree of local flexibility and fast motions on the picosecond to nanosecond time scale (39) consistent with unstructured regions. In contrast, positive {^1^H}-^15^N nOes reflect reduced flexibility indicative of structured regions (39). The {^1^H}-^15^N nOe for the bound DAD^M1199L^ showed that the structured region spans from G1197 to R1213 ({^1^H}-^15^N nOes >0.5), while the N and C termini are flexible (Fig. 2*A*). Importantly, bound DAD^M1199L^ exhibited increasingly negative {^1^H}-^15^N nOes from the positively charged RRKR motif (residues 1213–1216) to the C terminus.

### C terminus of DID IH has two conformations

The ^1^H-^15^N HSQC, spectrum of [*U*-^15^N]-DID in the presence of unlabeled DAD^M1199L^, is shown in Figure 1*C*. Two hundred twenty-five out of 234 (96%) residues were assigned. Importantly, the M1199L mutation resulted only in minor changes in the chemical shifts of DID, suggesting no major structural changes compared to the WT complex (Fig. S2). Cross peak doubling was observed for 13 C-terminal cross peaks of IH (Fig. 1*C* blue labels). {^1^H}-^15^N nOes acquired for [*U*-^15^N]-DID indicate that the C-terminal residues of IH conformer A exhibit restricted ps-ns dynamics suggesting that conformer A is structured (Fig. 2*B*). The C-terminal residues of IH conformer B exhibit increased ps-ns dynamics, suggesting that conformer B is unstructured (Fig. 2*B*). To assess the relative stability of these conformations, van’t Hoff analyses of selected C-terminal residues of IH were performed by collecting ^1^H-^15^N HSQC spectra of [*U*-^15^N]-DID bound to DAD^M1199L^ over the temperature range 280 to 310 K. Cross peaks G369, S373, Y374, and G378 were quantified and equilibrium constants for specific residues, K_eq_, were estimated as the ratio of the amide peak amplitudes of IH conformer A over IH conformer B (Fig. 2*C*). The negative slopes in Figure 2*C* showed that the unstructured to structured transition is endothermically unfavored and the *y*-intercepts in Figure 2*C* showed that the unstructured to structured transition is entropically favored. When combined, the IH conformer A trends to be thermodynamically unfavored over IH conformer B (Table. S1). The enhanced ps-ns dynamics of IH conformer B compared to conformer A, which results in a decrease in entropy, is likely compensated by the entropically favorable release of bound water, bound ions, or the structural rearrangement of other parts of DID during the B to A transition.

### Solution structure of the DID–DAD^M1199L^ complex

The solution structure of a 1:1 complex of DID-DAD^M1199L^, restricted to IH conformer A of DID, was resolved by using structural restraints derived from solution NMR and cross-linking MS (Figs. 1 and 2*D*, and Table S3). A standard suite of NMR experiments (38) was performed to assign backbone and side chain resonances of [*U*-^15^N,^13^C]-DID and [*U*-^15^N,^13^C]-DAD^M1199L^. To facilitate unambiguous assignment of DID residues, ^1^H-^15^N HSQC and 3D ^1^H-^15^N NOESY (38) spectra of individual ^15^N-labeled amino acids of DID bound to WT DAD were recorded at 305 K or 298 K. Intramolecular and intermolecular nOes from 3D ^1^H-^15^N NOESY spectra were collected for the DID–DAD^M1199L^ complex and converted into upper limits for the proton–proton distances utilized in structural calculations. Distance constraints from MS analyses of DID cross linked to DAD^M1199L^ were obtained by using amine-to-amine chemical cross-linkers. The cross-linked peptides were purified using SDS-PAGE and analyzed using tandem mass spectrometry (MS/MS). Cross-linkers of different lengths gave results for the same set of amines, indicating a flexible and transient structure of the N and C terminus, including the RRKR motif of DIAPH1. (Fig. S3 and Tables S3 and Fig. S4). For this reason, our structure calculations used cross-links that provided tighter distance constraints. A typical spectrum is shown in Figure 2*D* for the cross-linked product between the N terminus of DAD^M1199L^ and K228 of DID.

**Figure 3:**
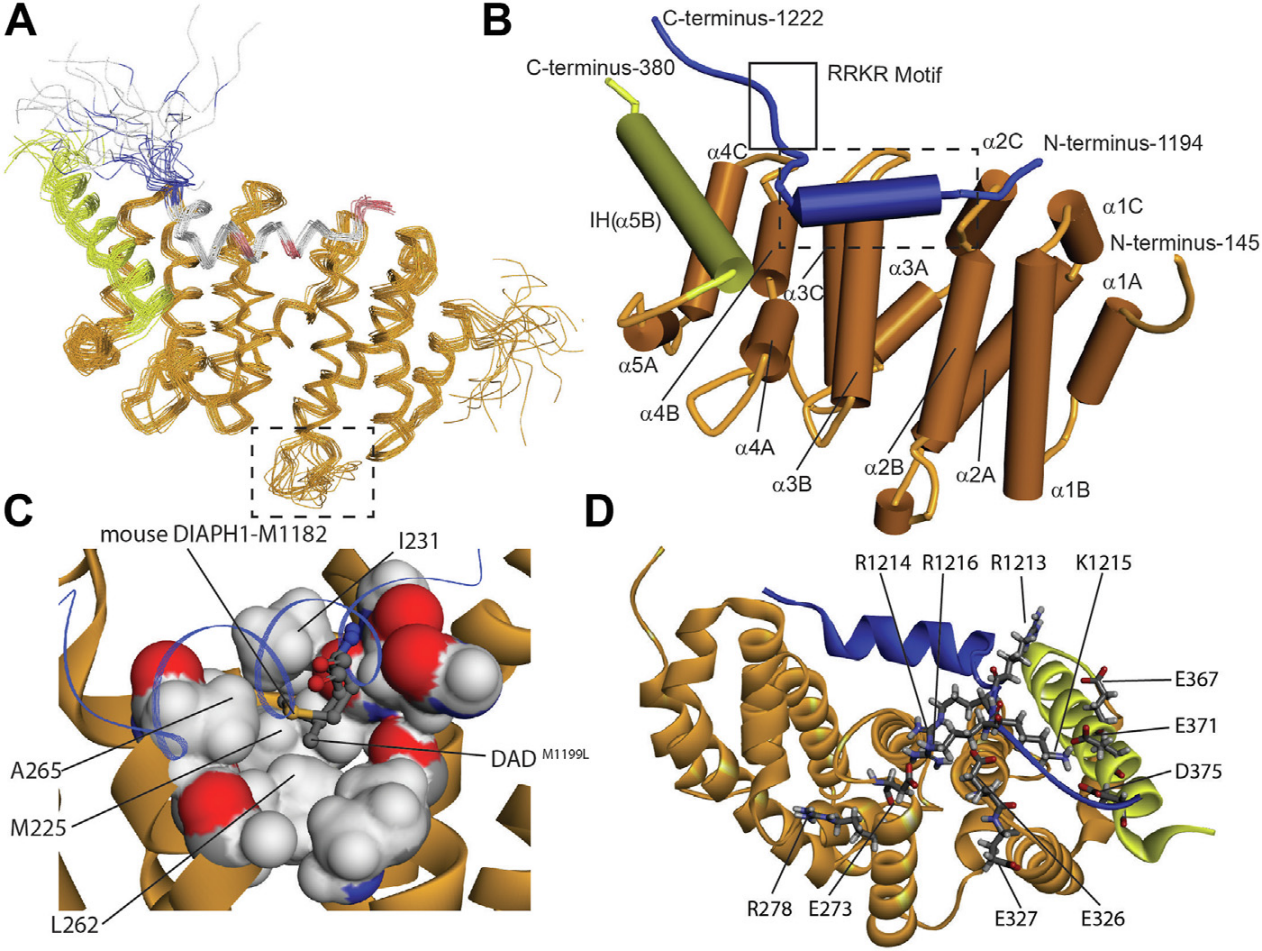
**Solution structure of human DIAPH1 DID–DAD**^**M1199L**^**complex reveals the binding mode of the RRKR motif.***A*, overlay of the backbone traces of the 20 solved DID-DAD^M1199L^ solution structures (PDB code 8FG1) refined using YASARA (42). The ARR region of DID is in *orange*, the IH is in *yellow*, and the DAD^M1199L^ peptide is colored by pKa where *blue* is basic, *gray* is neutral, and *red* is acidic. The *dashed box outlines* residues absent from published structures. *B*, *tube diagram* of a representative DID-DAD^M1199L^ structure showing the superhelical orientation of the armadillo repeats of DID and the DAD^M1199L^ peptide (*blue*). The *dashed box outlines* in *panel C*. The *solid box outlines* the RRKR motif in *panel D*. *C*, close up of the DID van der Waals surface of residues within 5 Å of L1199. *White* is hydrogen, *red* is oxygen, *blue* is nitrogen, *gray* is carbon, and *orange* is sulfur. M1182 of mouse DIAPH1, which corresponds to M1199 of DIAPH1, and L1199 side chains are shown as *balls* and *sticks*. The DAD^M1199L^ peptide backbone (this work) is shown as a *thin blue ribbon*. M1182 is from PDB 2F31. *D*, DID–DAD^M1199L^ complex, rotated 180^°^ horizontally and downward 90^°^ relative to 3B, showing putative interactions between the basic RRKR motif and acidic residues of DID. Residues E273, R278, E367, E371, and D375 were subjects of previous mutagenesis studies (23, 25). All models were created in Discovery Studio. ARR, armadillo repeat region; DAD, diaphanous autoinhibitory domain; DIAPH1, Diaphanous 1; DID, diaphanous inhibitory domain; IH, interdomain helix.

Backbone chemical shifts of DID and DAD^M1199L^ were used to predict acceptable ranges of backbone torsion angles per residue using Torsion Angle Likelihood Obtained from Shift and sequence similarity (TALOS)-N software (40). Structural calculations were performed by using Combined assignment and dYnamics Algorithm for NMR Applications (CYANA) (41), followed by refinement using Yet Another Scientific Artificial Reality Application (YASARA) (42) in combination with 901 DID intramolecular nOes, 94 DAD^M1199L^ intramolecular nOes, 13 DID-DAD^M1199L^ intermolecular nOes, (Table S2), two DID intramolecular cross-link distances, and two DID-DAD^M1199L^ cross-link distances (Table S3, and Fig. S3). The torsion angles were combined with the distance restraints to yield an ensemble of 20 solution structures for the DID–DAD^M1199L^ complex with a backbone RMSD of 0.3 Å for the helical regions (Fig. 3*A*), indicating high quality structural convergence. Notably, the loop between α2A and α2B, which is absent from the published structures, was well-resolved despite some conformational variability (Fig. 3*A*, dashed box).

The core of DID presents a five-armadillo repeat arranged in a superhelical coil (Fig. 3*B*). Despite eleven sequence alterations compared to mouse DIAPH1, P160H, S206A, N208S, Q215K, S307T, E349D, K359R, C363N, D370E, F373S, and F374Y, there were no significant deviations of the ARR from previously published mouse DIAPH1 structures (11, 15, 16, 17, 23, 25, 43, 44). Superposition of the ARR in the DIAPH1 DID-DAD^M1199L^ solution structure and mouse DIAPH1 crystal structure (16) yielded a backbone RMSD of ∼1 Å.

DAD^M1199L^ peptide residues V1198-S1208 formed an amphipathic α-helix that was shorter than previously resolved for mouse DIAPH1 (17, 43) and contacted the concave hydrophobic surface comprised of the central B helices in DID (Fig. 3*B*). Residues G1209-R1213 turned perpendicular to the helix, anchoring at F1212 (F1195 in mouse DIAPH1), in agreement with published structures (11, 15, 16, 17), while the positively charged C terminus remained unstructured. The L1199 side chain had a dihedral angle of χ_1_ = −160^°^, comparable to the −177^°^ observed for the corresponding residue, M1182, in the crystal structure of the complex formed by the C- and N-terminal fragments of mouse DIAPH1 (16). L1199 also has similar χ_2_ and χ_3_ values and resides in a hydrophobic pocket contacting M225, I231, L262, and A265, in agreement with published structures (Fig. 3*C*) (11, 15, 16, 17) The results confirmed the NMR observations (Fig. S2) and demonstrated that the leucine substitution does not perturb the WT structure.

Encounter complexes are reaction intermediates that arise from an ensemble of conformations that can sample various orientations to properly align binding determinants (33, 34, 45). As postulated for other cases of encounter complexes (33, 34, 45), DAD residues R1213-R1216 are electrostatically steered (34, 45) toward the negatively charged surface of DID. The RRKR motif does not exhibit NMR–visible interactions with DID and the entirety of this highly basic region is absent in the published structures. Cross-linking together with MS/MS experiments showed that DAD^M1199L^ K1215 is within 11.4 Å of DID K228 and 11.4 Å of DID K377 (Table S3). In addition, MS analysis yielded cross-links for the K228-K1215 pair obtained using a 21.7 Å linker (Table S4). Both DID lysine residues are too far apart to interact simultaneously with DAD^M1199L^ K1215, indicating a high degree of flexibility for the C terminus of DAD^M1199L^ in agreement with the NMR data. When either the DAD^M1199L^ K1215/DID K377 or DAD^M1199L^ K1215/DID K228 cross-link was used for structure calculations, the RRKR motif interacted with the acidic DID residues E326, E367, E371, and D375, in agreement with mutagenesis studies (23, 25) (Figs. S4 and 3*D*). These residues constitute a negative patch on DID that interacts with the basic RRKR motif of DAD^M1199L^, suggesting that electrostatic steering may play a role in complex formation as previously described for productive encounter complexes (33, 34, 45, 46).

### T-helix does not impede DID–DAD^M1199L^ interactions

Available structures of DRFs revealed a long helical structure preceding DAD^M1199L^, the T-helix, which also contacts DID (15, 17). This helix was poorly resolved in the crystal structure suggesting flexibility (15, 17). We used NMR spectroscopy to resolve this element and observed a well-dispersed ^1^H-^15^N HSQC spectrum of the T-helix, which indicated the presence of a folded structure (Fig. S5*A* and Table S5). Analyses of amide–amide cross-peaks in the ^1^H-^1^H NOESY spectrum (Fig. S5*B*) and chemical shift indices (Fig. S5*C*) revealed a transient helical structure in solution. Superimposing the DID-DAD^M1199L^ structure from this work with the T-helix and DAD from mouse DIAPH1 (15) shows that the RRKR motif of DAD^M1199L^ appears to sterically clash with the T-helix (Fig. S5*D*). Such an occlusion could prevent the formation of an encounter complex by masking the acidic patch on DID. Contribution of the RRKR motif to the overall binding affinity of DID for DAD^M1199L^ can be estimated from binding studies^11^ to be in the millimolar range, assuming that binding of the DAD helix and the RRKR motif to DID are cooperative. To probe this interaction, 120 μM of [*U*-^15^N]-DAD^M1199L^ bound to unlabeled DID was titrated with 360 μM of purified T-helix and analyzed *via* NMR (Fig. S5*E*). The absence of chemical shift perturbations in [*U*-^15^N]-DAD^M1199L^ indicates that there are no T-helix residues that compete with RRKR for binding to DID.

### Double mutant DID^E326G/E327A^ alters the affinity and kinetics of DAD^M1199L^ binding

**Figure 4:**
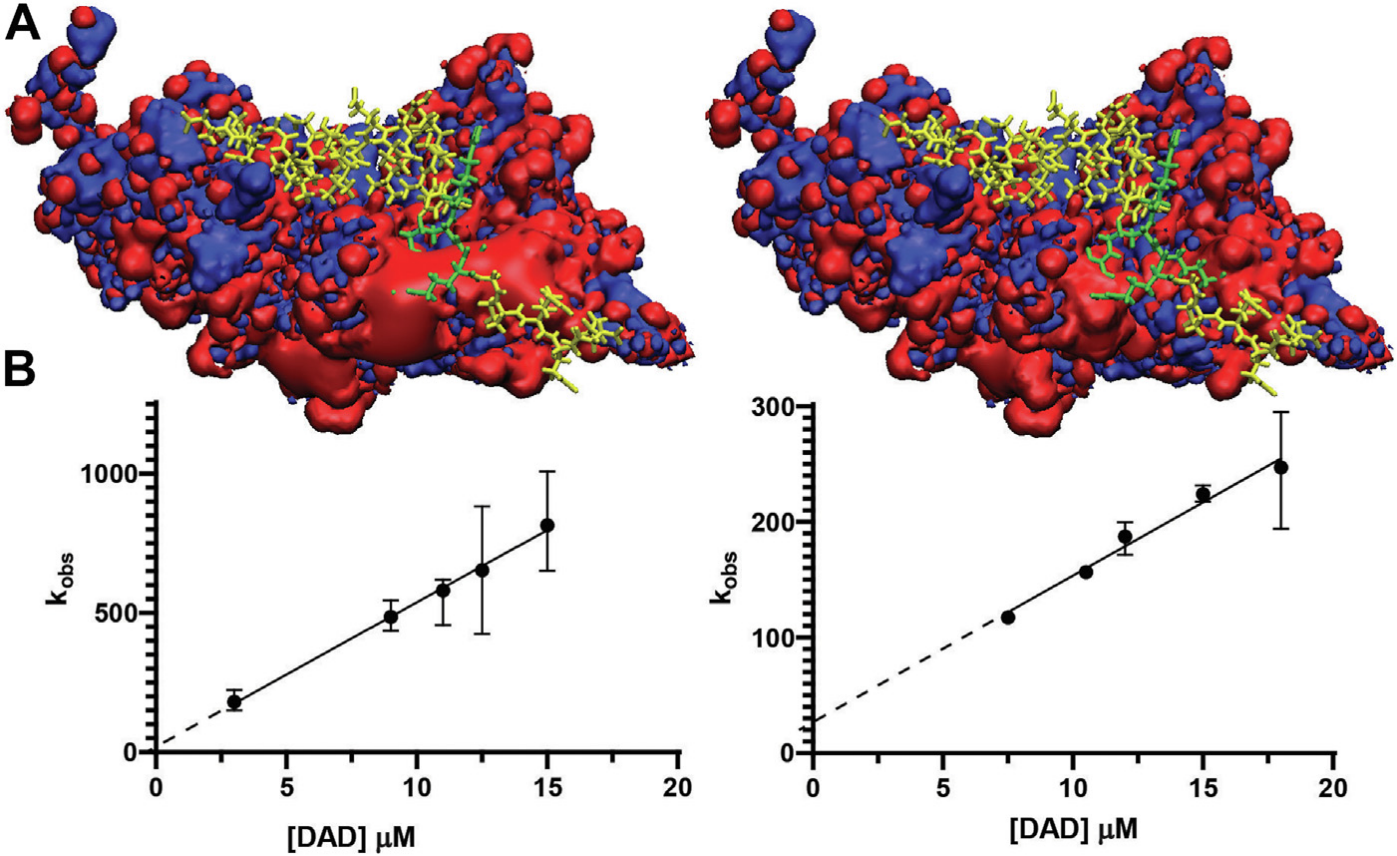
**The acidic patch of DID is required for strong DAD**^**M1199L**^**binding.***A*, iso-potential surface of DID (*left*) and DID^E326G/E327A^ (*right*) bound to DAD^M1199L^ where *blue* and *red* represents +10 eV and −10 eV, respectively. The basic RRKR motif of DAD^M1199L^ is in *green* and the rest of DAD^M1199L^ is in *yellow*. The E326G and E327A mutations reduce a large negatively charged surface on DID as evidenced by the relocation of the basic side chain (*green*) above the −10 eV iso-contour surface (*right*). The orientation is analogous to Figure 3*D*. *B*, stopped flow fluorescence titrations for the association kinetics of DID-DAD^M1199L^ (*left*) and DID^E326G/E327A^-DAD^M1199L^ (*right*). Surface models of DID and residues of DAD^M1199L^ were made by using Visual Molecular Dynamics software (65). All graphs were made using GraphPad Prism (graphpad.com). DAD, diaphanous autoinhibitory domain; DID, diaphanous inhibitory domain.

To biochemically test if the acidic patch on DID (Fig. 3*D*) is involved in electrostatic steering (34, 45), mutations E326G/E327A, DID^E326G/E327A^, were introduced. These mutations change the electrostatic character of the patch converting it from negatively charged to mostly neutral (Fig. 4*A*). The ^1^H-^15^N HSQC spectrum of [*U*-^15^N]-DID^E326G/E327A^ complexed with DAD^M1199L^ exhibited a well-dispersed amide envelope and minor chemical shift changes (Fig. S6, *A* and *B*) as compared to that of the WT [*U*-^15^N]-DID–DAD^M1199L^ complex, indicating that the tertiary structure of DID^E326G/E327A^ is comparable to that of the WT.

To test if the DID negative patch is critical for DAD binding, tryptophan fluorescence titrations were performed with 200 nM DID^E326G/E327A^ and DAD^M1199L^ concentrations ranging from 10 to 90 μM (Fig. S1*C*). The K_D_ resolved for DID^E326G/E327A^ was 1.5 ± 0.5 μM, which is about five times higher than that for WT DID (Fig. S1*B*). This result suggests that the interaction between the RRKR motif of DAD^M1199L^ and the negatively charged patch of DID is specific and increases the overall affinity of DAD^M1199L^ for DID.

Stopped flow fluorescence titrations were used to evaluate the effect of the E326G/E327A mutation on the DID-DAD^M1199L^ association kinetics. Two hundred nanomolar (200 nM) DID and DID^E326G/E327A^ were titrated with 1 to 20 μM DAD^M1199L^ to create pseudo first order kinetics. The data were fit to a single exponential to estimate the observable rate constants, k_obs_ (Fig. 4*B*). A k_on_ of 52 ± 1.5 (μM s)^−1^ and a k_off_ of 19 ± 17 s^−1^ were resolved for the DID–DAD^M1199L^ interaction and a k_on_ of 13 ± 1 (μM s)^−1^ and a k_off_ of 27 ± 14 s^−1^ for the DID^E326G/E327A^–DAD^M1199L^ interaction. The 4-fold decrease in k_on_ suggests that the decreased binding affinity of DAD^M1199L^ to DID^E326G/E327A^ is primarily due to a decrease in the rate of association, in agreement with an electrostatic steering mechanism.

### DAD^M1199L^ also forms futile encounter complexes by nonspecific binding to DID

**Figure 5:**
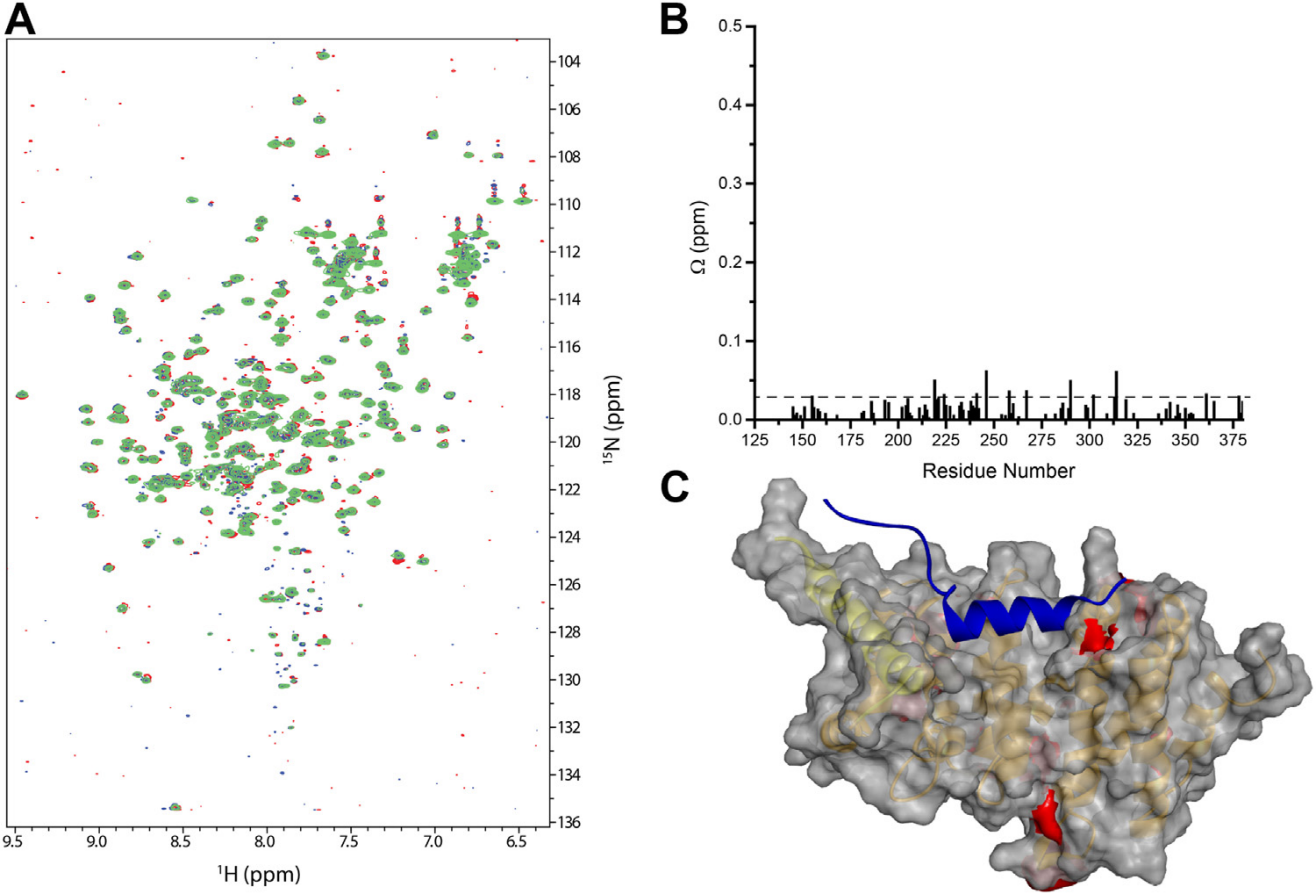
**Nonspecific binding of DAD**^**M1199L**^**results in the formation of futile encounter complexes.***A*, superimposed ^1^H-^15^N-HSQC spectra of 100 μM [*U*-^15^N] DID in the presence of a 1× (*red*), 10× (*blue*), and 18× (*green*) molar excess of DAD^M1199L^. *B*, chemical shift changes, Ω, in the ^1^H-^15^N-HSQC spectra of [*U*-^15^N]-DID in the presence of a 1× and 18× molar excess of DAD^M1199L^. Ω = √((ΔH)^2^ + (ΔN/2)^2^), where ΔH and ΔN are changes in chemical shift in the hydrogen and nitrogen dimensions, respectively. *C*, semitransparent space filling model of DID showing significant chemical shift changes (*red*) upon nonspecific DAD^M1199L^ binding. The cut-off value for significant changes, Ω = 0.03, defines the largest 5% of the Ω values. The core structure of DID is *tinted brown* and the interdomain helix is *yellow*. The orientation of the DAD^M1199L^ peptide in the encounter complex is shown in *blue*. DAD, diaphanous autoinhibitory domain; DID, diaphanous inhibitory domain; HSQC, heteronuclear single–quantum coherence.

The model used to fit the tryptophan fluorescence data (Fig. S1) could not discriminate between specific and nonspecific interactions. To identify residues involved in nonspecific interactions, [*U*-^15^N]-DID was titrated with DAD^M1199L^ and changes in the ^1^H-^15^N HSQC spectra were monitored (Fig. 5*A*). With a large molar excess of DAD^M1199L^, nonspecific binding interactions could be inferred from changes in DID chemical shifts. We observed minor chemical shift perturbations of DID residues (Fig. 5*B*), most of which were clustered in at least four noncontiguous areas on the DID surface (Fig. 5*C*). Thus, our chemical shift mapping reveals nonspecific binding sites for DAD^M1199L^ that could give rise to so-called futile encounter complexes (34).

### Mutations in the negative patch of DID result in dysregulation of DIAPH1 activity

Autoinhibited DIAPH1 is free and cytosolic; however, upon activation it binds to the ends of actin fibers (3, 4). Deletion of the DAD sequence results in activation of DIAPH1 and increased actin binding (11, 22, 23). Importantly, deletion of the RRKR motif in DIAPH1 also results in elongation of microvilli with subsequent increased DIAPH1-actin colocalization (24). We hypothesized that mutations E326G/E327A in DID, which disrupt the binding of RRKR and thus the formation of the productive encounter complex, would also lead to increased DIAPH1-actin colocalization.

**Figure 6:**
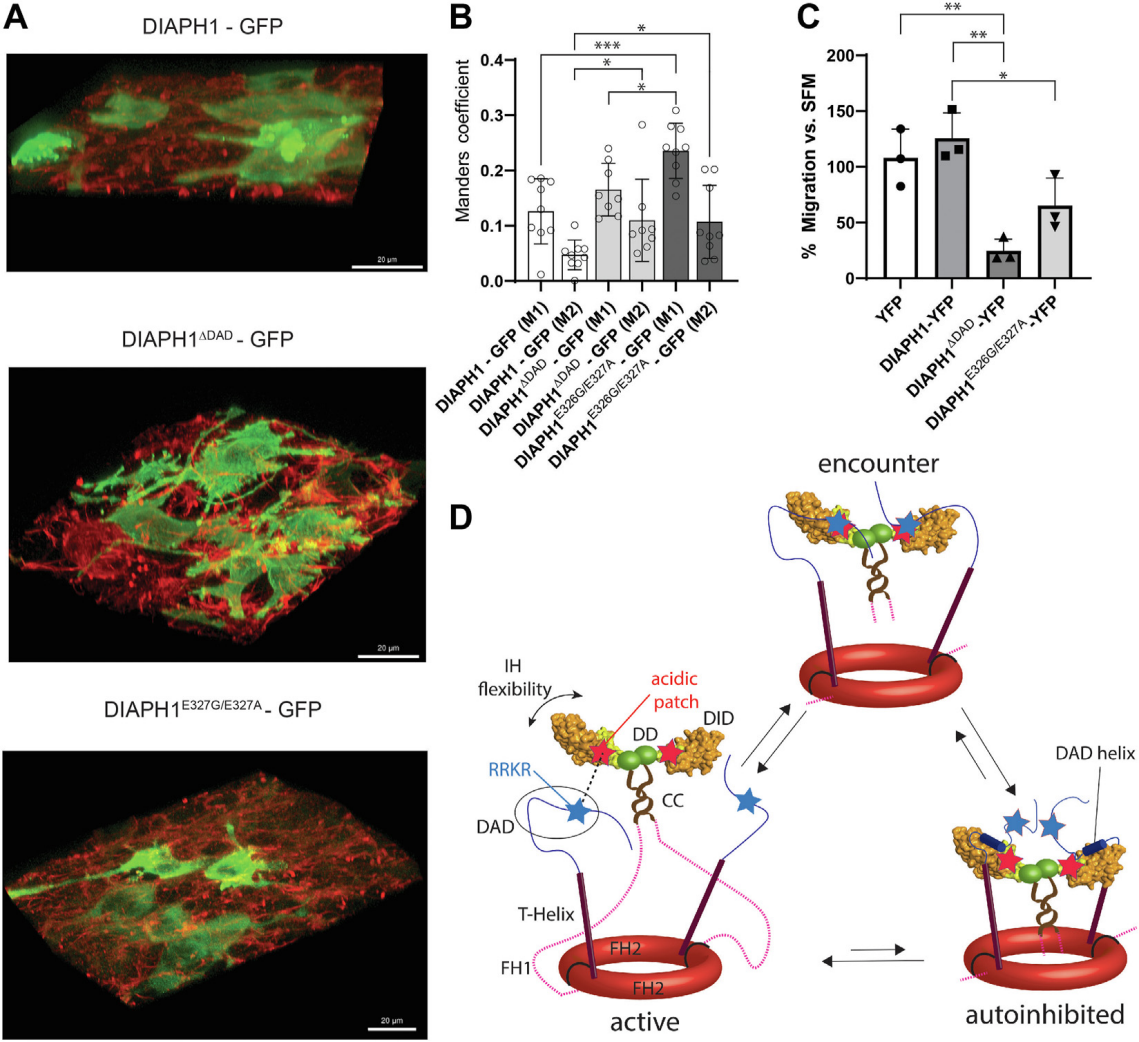
**Formation of a productive encounter complex increases DIAPH1-actin colocalization and regulates RAGE ligand–induced cellular migration in human vascular smooth muscle cells.***A*, fluorescence microscopy of HEK293T cells with GFP-tagged WT DIAPH1 (*top*), DIAPH1^ΔDAD^ (*middle*), and DIAPH1^E326G/E327A^ (*bottom*) in *green*, and actin labeled with Alexa Fluor 568 Phalloidin in *red*. *Yellow* indicates colocalization. *B*, manders coefficients, M1 and M2, quantifying colocalization between F-actin and DIAPH1 constructs and DIAPH1 constructs and F-actin, respectively. Larger values correspond to a higher degree of colocalization. Column heights reported on the graph represent mean values, error bars represent the SD. *C*, percent migration of human primary aortic vascular smooth muscle cells, SMCs, when stimulated with CML-HSA as compared to those treated with serum-free medium (SFM). Cells expressing YFP and DIAPH1-YFP were used as a reference and compared with those expressing DIAPH1^ΔDAD^-YFP and DIAPH1^E326G/E327A^-YFP. *Stars* indicate statistical significance: *p*-value <0.05 (∗), *p*-value <0.01 (∗∗), and *p*-value <0.001 (∗∗∗). *D*, proposed model for the equilibrium of WT DIAPH1 dimers between the active, encounter, and autoinhibited complexes. *Double headed arrow* indicates IH flexibility, and unstructured DAD is shown in the *circle*. Colored domains match that of Figure 1*A*. In mouse DIAPH1, DD, CC, and FH2 domains facilitate dimerization (15, 17, 43, 72). Active DIAPH1 forms a productive encounter complex *via* electrostatic steering, *dotted line*, between the RRKR motif (*blue star*) and the acidic patch of DID (*red star*). The interaction increases the probability of DID-DAD helix coupled folding and binding and formation of the autoinhibited conformation. Active DIAPH1 lacking the basic RRKR motif, such as in DIAPH-(R1213X) (22, 31), cannot create a productive encounter complex resulting in an equilibrium shift toward the active complex. Note that deletion of DAD, such as in DIAPH1^ΔDAD^, would preclude the autoinhibition. CC, coiled-coil; CML-HAS, carboxymethyllysine human serum albumin; DAD, diaphanous autoinhibitory domain; DIAPH1, Diaphanous 1; DID, diaphanous inhibitory domain; IH, interdomain helix; RAGE, receptor for advanced glycation end products; YFP, yellow fluorescent protein.

To test this hypothesis, the DAD deletion mutant (12) (DIAPH1^ΔDAD^), which was used as a positive control, and double mutant DIAPH1^E326G/E327A^, were introduced into HEK293T cells. All constructs were expressed with an N-terminal GFP tag to monitor the location of the proteins by fluorescent microscopy. F-actin was tagged with phalloidin-Alexa Fluor 568 to measure the colocalization of DIAPH1-GFP constructs with F-actin (Fig. 6*A*). Manders coefficients, M1 and M2 (47), were used to quantify the colocalization of F-actin with DIAPH1-GFP constructs and DIAPH1-GFP constructs with F-actin, respectively (Fig. 6*B*). As expected, both DIAPH1^ΔDAD^-GFP and DIAPH1^E326G/E327A^-GFP exhibited significant increases in colocalization with F-actin compared to that of the WT.

To further test the functional importance of the RRKR motif, we examined RAGE signal transduction (7, 8, 9). DIAPH1 is an intracellular effector of RAGE; stimulation of RAGE by RAGE ligand AGEs, such as carboxymethyllysine human serum albumin (CML-HSA) results in a DIAPH1-dependent increase in migration of vascular smooth muscle cells (SMCs) (7, 8, 9). Human SMCs were transfected with plasmids-expressing yellow fluorescent protein (YFP), DIAPH1-YFP, DIAPH1^ΔDAD^-YFP, and DIAPH1^E326G/E327A^-YFP, and the cells were treated with 1 μM of CML-HSA (Fig. 6*C*). SMCs transfected with DIAPH1-YFP exhibited an insignificant increase in migration upon stimulation compared to those transfected with YFP, suggesting that endogenous DIAPH1, which is present in SMCs, is sufficient to support RAGE-dependent cell migration. SMCs transfected with the positive control DIAPH1^ΔDAD^-YFP exhibited a statistically significant reduction in cell migration upon stimulation, suggesting that the deletion of DAD disrupts RAGE signal transduction. SMCs transfected with DIAPH1^E326G/E327A^-YFP also exhibited a statistically significant reduction of cell migration upon stimulation, although this reduction trended lower than that of the positive control.

## Discussion

The solution structure of human DID-DAD^M1199L^ elucidated features of autoinhibited human DIAPH1 that were unresolved in previous crystal structures of the mouse analog to reveal important aspects of DIAPH1 dynamics and its role in regulation. Part of the IH of DIAPH1, residues 367 to 380, exhibits an equilibrium between structured and unstructured conformers. The structured conformation is helical and similar to that detected in crystalline mouse DIAPH1 DID (11, 15, 16, 17, 23, 25, 43, 44). Thermodynamic data show that the structured form is likely favored but still retains enough flexibility observed in variability between DID and DD domains in crystalline forms of mouse DIAPH1 (11, 15, 23, 25). Flexibility in the regions between the DID and the rest of DIAPH1 contributes to the formation of the autoinhibited state by increasing the probability of DID, encountering DAD in the context of full-length DIAPH1.

The lack of structural information on the DAD C terminus complicates efforts to understand atomic-level interactions between the basic RRKR motif and DID implicated in human pathologies (24, 29, 30, 32). Previous studies have shown that the RRKR motif is not essential for DID–DAD binding but does contribute to the binding affinity (11, 22, 31). Removal of the RRKR motif decreases DID-DAD binding affinity by 5-fold (11), resulting in gain-of-function (24). Our NMR and cross-linking studies show that the RRKR motif is flexible in the DID–DAD^M1199L^ complex (Tables S3 and S4). How could such a strong effector of K_D_ be flexible? Findings from cross-linking and stopped flow experiments in this work reveal transient, yet specific, interactions involving RRKR, thus providing insight into a possible structural mechanism of autoinhibitory regulation.

Earlier structural studies revealed that Rho GTPase binding to DIAPH1 elicits a conformational change that displaces the DAD helix from DID (12, 25, 48). These studies showed that the binding causes only partial activation of DIAPH1, suggesting that other regulatory steps are involved in DIAPH1 activity. Our study further elaborates on this point by at least partially implicating the formation of an endogenous encounter complex in the regulation of DIAPH1 activity.

Previous mutagenesis studies showed that mutating DID residues E367R, E371R, or D375R (23), which are located in the vicinity of the RRKR-binding site (Figs. 3*D* and S4), resulted in a 2- to 3-fold decrease in DID-DAD binding affinity, but E273K and R278E did not (25). Our findings identify E326 and E327 as critical for binding (Figs. 3*D*, 4 and S1). The E326 and E327 acidic patch is located greater than 30 Å away from the Rho GTPase binding site and represents a new surface for DIAPH1 regulation (Fig. S4*B*) (25). Interruption of the electrostatic interaction between the basic RRKR motif and these acidic residues decreased DID-DAD^M1199L^ binding affinity by a factor of five. Due to the long-range nature of electrostatic interactions, the combined effect of E326G/E327A mutations with those previously studied (23) is expected to be cumulative. Importantly, the E326G/E327A mutations that disrupt the RRKR binding surface also increased colocalization of actin and DIAPH1 *in vivo* (Fig. 6, *A* and *B*), consistent with previous observations that deletion of RRKR leads to DIAPH1 colocalization with actin in filopodia (24).

DIAPH1 is critically involved in RAGE signaling: SMC migration is increased when RAGE is activated by its ligand CML-HSA and this increase depends on RAGE-DIAPH1 signal transduction (8, 9, 10). Perhaps counterintuitively, the E326G/E327A mutation resulted in significant reduction of SMC migration. Note that efficient cellular migration relies on regulation of actin polymerization/depolymerization and, thus, dysregulation leads to reduced migration (49, 50, 51). This suggests that DIAPH1^E326G/E327A^ was not fully regulated by autoinhibition (Fig. 6*C*). An even larger reduction was observed in the SMCs expressing DIAPH1^ΔDAD^ where the regulatory DID-DAD interaction is completely removed; DIAPH1^ΔDAD^ was used as a positive control for dysregulated constitutively active DIAPH1.

Because the K_D_ for DID-DAD^M1199L^ increased when DID E326G/E327A mutations were introduced and the interaction remained transient, it is likely that DID^E326G/E327A^ mutations affect primarily the association of DAD^M1199L^ to DID. Kinetic experiments and NMR titrations suggest that RRKR interacts with the acidic residues on DID and this interaction is not obstructed by the T-helix (15, 17). Altogether, these observations paint a dynamic picture. The conformational exchange between structured and unstructured in the IH segment of DID allows the DID to search more conformational space. This, in turn, allows the basic RRKR motif to interact with the acidic patch of DID facilitating the wobbling and searching (52), folding and binding (53) of the DAD helix. This interaction is absent in RRKR deletion mutants leading to shifting equilibrium to the active form of DIAPH1 (Fig. 6*D*) (22, 31). Subsequent exchange of RRKR with the basic portion of the T-helix may then free the C terminus of DAD. This agrees with the current understanding that the RRKR motif interacts with actin and facilitates actin fiber nucleation (22, 54).

Importantly, the mechanism proposed here describes a productive encounter complex between DID and DAD required for autoinhibition, which can be disrupted by mutations in DID or deletions in DAD leading to gain-of-function activity. This suggests that the disease states characterized by gain of function, such as autosomal dominant hearing loss (24) and macrothrombocytopenia (32), may arise from enhanced actin assembly mediated by DIAPH1 dysregulation.

## Experimental procedures

### DID cloning, expression, and purification

Expression plasmid pET-28a(+)-DID, which confers kanamycin resistance and expresses an N-terminal his-tagged DID, residues 142 to 380 from human DIAPH1 (UniProt O60610), was purchased from Genscript. pET28a(+)-DID was transformed into *Escherichia coli* strain BL21(DE3) and grown overnight at 37 °C in 100 ml of LB containing 35 μg/ml of kanamycin (LB-Kn). The culture was transferred to 1 L of LB-Kn and incubated at 37 °C until the *A*_550_ reached 0.90. To overexpress DID without stable isotope labeling, IPTG was added to a final concentration of 1 mM and the cells were incubated for 4 h at 37 °C. For isotopic labeling, cells were harvested by centrifugation at 4000*g* and resuspended in minimal (M9) medium, 5 mM Na_2_HPO_4_, 2.5 mM KH_2_PO_4_, pH 7, 1 mM NaCl, 2 mM MgSO_4_, 0.1 mM CaCl_2_, and 1 mg/l thiamine hydrochloride, supplemented with 35 μg/ml of kanamycin (M9-Kn). For [*U*-^15^N] labeling, 1 g/l of ^15^N-ammonium chloride (Sigma-Aldrich) was used as the sole nitrogen source. For [*U*-^15^N,^13^C] labeling 2 g/l of ^13^C-glucose (Sigma-Aldrich) was used as the sole carbon source. Overexpression of labeled DID was induced with 1 mM IPTG and proceeded for 4 h at 37 °C. Cells were centrifuged at 4000*g*, resuspended in lysis buffer, 20 mM Hepes, pH 8, 1 M NaCl, 12.5% w/v sucrose, 4 M urea, and 10 mM β-mercaptoethanol, containing an EDTA-free protease inhibitor tablet (Roche) and sonicated with a Model 250 Digital Sonifier (Branson) at 40% amplitude using 2.5-min cycles of 0.3 s pulses interrupted by 1 s pauses. The lysate was centrifuged for 40 min at 20,000*g* and 4 °C. The supernatant was loaded into a nickel-nitriloacetic acid agarose affinity column (Qiagen) pre-equilibrated with lysis buffer. The partially denatured protein bound to the column was refolded by washing with five column volumes of refolding buffer, 10 mM Hepes, pH 8, 300 mM NaCl, and 10 mM imidazole. The column was washed with five column volumes of wash buffer, 10 mM Hepes pH 8, 300 mM NaCl, and 20 mM imidazole, and the refolded DID was eluted with elution buffer, 10 mM Hepes, pH 8, 300 mM NaCl, and 250 mM imidazole. EDTA was added from a 0.5 M pH 8 stock to a final concentration of 10 mM, and the eluant was dialyzed into ion exchange chromatography buffer A, 20 mM NaH_2_PO_4_/Na_2_HPO_4_, pH 8.10, 100 mM NaCl, prior to loading onto a Hi-Trap Q-XL anion exchange column (Cytiva). A linear gradient of buffer B, 20 mM sodium phosphate, pH 8.10, 1 M NaCl, was used to elute the protein at a conductivity of ∼40 mS/cm. The purity of the DID protein (molecular weight of 29,078 Da) was found to be >95% on a 12% SDS-PAGE, and the concentration was determined by absorbance using a molar extinction coefficient of 10,220 M^−1^ cm^−1^ at 280 nm.

DID^E326G/E327A^ was overexpressed using the plasmid pET-28a(+)-DID ^E326G/E327A^ (GenScript), which confers kanamycin resistance and expresses an N-terminal his-tagged, mutated DID^E326G/E327A^. Purification was performed using the protocol identical to that of DID.

Residue-selective labeling of DID was accomplished by transferring the bacterial cells grown in 1 L of LB-Kn to 1 L of M9-Kn medium containing a mixture of 19 unlabeled amino acids plus 0.5 g/l of the ^15^N-labeled amino acid of interest (55). Overexpression was carried out for 4 h at 21 °C after adding IPTG to 1 mM. The cells were harvested and selectively labeled DID protein was purified using the methodology described above for [*U*-^15^N]-labeled DID.

### DAD and DAD^M1199L^ cloning, expression, and purification

The 29-residue DAD peptide, DETGVMDSLLEALQSGAAFRRKRGPRQAN, corresponding to WT residues 1194 to 1222 (UniProt O60610), was purchased from GenScript. Stock solutions were prepared by dissolving the peptide in 10 mM potassium phosphate, pH 6.8 containing 15 mM hexamethylphosphoramide, HMPA. Concentrations were calculated using the dissolved weight of the lyophilized peptide.

The DNA sequence for the M1199L mutant peptide, DAD^M1199L^, was purchased from GenScript and inserted into pTM-7 to express an N-terminal his-tagged TrpL-DAD^M1199L^ fusion protein (35). Fusing the DAD^M1199L^ peptide to a hydrophobic protein (TrpL) directed the gene product into inclusion bodies. The methionine residues in TrpL were mutated to leucine residues (36) to allow for a single cyanogen bromide cleavage site between TrpL and DAD^M1199L^. The plasmid was transformed into *E. coli* BL21(DE3) to overexpress both labeled and unlabeled peptide as described for the DID constructs.

To purify DAD^M1199L^, the bacterial cell pellet was resuspended in 50 mM Tris–HCl, pH 7.2, 1% w/v Triton X-100, and 1 mM EDTA, sonicated as described above and centrifuged at 10,000*g* for 30 min at 4 °C. The pellet was washed with a series of buffers, 50 mM Tris–HCl, pH 7.2, containing 2% w/v Triton X-100 and 1 mM EDTA, followed by 25 mM Tris–HCl, pH 7.2 containing 1 M NaCl and 0.5 mM EDTA, and 50 mM Tris–HCl, pH 7.2. The cells were sonicated and centrifuged between washes. The final pellet was dissolved in denaturing buffer, 50 mM sodium phosphate, pH 8.3 containing 6 M guanidinium chloride, and clarified by centrifugation at 10,000*g*. The supernatant was incubated with nickel-nitriloacetic acid agarose beads overnight for batch binding. The beads were packed into a column and washed once with denaturing buffer. The column was washed with five volumes of 50 mM sodium phosphate, pH 7 containing 6 M urea, followed by 50 mM sodium phosphate, pH 6.4, and 6 M urea. Elution was carried out using 50 mM sodium phosphate, pH 3.6, and 6 M urea. Fractions containing TrpL-DAD^M1199L^were pooled, dialyzed into water, and lyophilized. Cleavage was performed by dissolving the TrpL-DAD^M1199L^ fusion protein in 70% formic acid with a 100 M excess of cyanogen bromide and incubating for 1.5 h at 25 °C. The cleaved products were dried *in vacuo*, loaded onto a C18 column (Agilent ZORBAX 300SB-C18) for HPLC. Cleaved products were resolved using a gradient of 0% to 90% acetonitrile in 0.1% trifluoroacetic acid. DAD^M1199L^ eluted first and was collected, lyophilized, and stored at −20 °C. DAD^M1199L^ stock solutions were prepared by dissolving the peptide in 10 mM potassium phosphate buffer, pH 6.8 containing 15 mM HMPA. The concentration of DAD^M1199L^ stock solutions was determined by integrating HPLC chromatogram traces at 260 nm, the approximate wavelength of maximal absorption of phenylalanine residues relative to reference WT DAD peptide solutions.

### T-helix expression and purification

The T-helix protein, KRRETEEKMRRAKLAKEKAEKERLEKQQ, corresponding to residues 1154 to 1181 (UniProt O60610) was purchased from GenScript as a lyophilized powder. Samples were resuspended in 90 mM sodium phosphate, pH 7.2, 75 mM sodium chloride, 0.01% sodium azide, and 10%v/v D_2_O at 291 K for structure determination and used immediately for NMR experiments or stored at −20 °C until further use.

### Cross-linking reactions

Amine-to-amine cross-linking of unlabeled DID to DAD^M1199L^ was performed using disuccinimidyl glutarate (DSG), bis(sulfosuccinimidyl) suberate (BS3), and bis(succinimidyl) penta(ethylene glycol) (BS(PEG)_5_) purchased from Thermo Fisher Scientific. These amine-to-amine cross-linkers had spacer arm lengths of 7.7, 11.4, and 21.7 Å, respectively. Cross-linkers were added to an equimolar mixture of 200 μM DID/DAD^M1199L^, in cross-linking buffer, 20 mM potassium phosphate buffer, pH 7.5, 100 mM NaCl, to final concentrations of 2.5 mM for DSG and BS3, and 1 mM for BS(PEG)_5._ Cross-linking reactions were incubated for 2 h on ice or 30 min at 25 °C. Reactions were quenched by adding 50 mM Tris–HCl, pH 7.5 to a final concentration of 62 mM.

The cross-linked products were resolved by using 8% SDS-PAGE. Bands corresponding to cross-linked DID/DAD^M1199L^ were visualized by staining with Coomassie Blue R-250 (Fig. S3*B*). The bands of interest were excised from the gels and cut into millimeter-length pieces. The gel pieces were destained in 50% v/v methanol 5% v/v acetic acid, dehydrated by soaking in acetonitrile, and lyophilized. The gel pieces were treated with 10 mM DTT for 15 min at 37 °C to break disulfide bonds, then with 50 mM iodoacetamide for 30 min at 25 °C to alkylate the protein sulfhydryl groups. Excess iodoacetamide was removed by washing the gels with 100 mM NH_4_HCO_3_, followed by dehydrating in acetonitrile. After three rounds of washing and dehydrating, the gel pieces were completely dried by lyophilization. In-gel tryptic digestion was performed by incubating the lyophilized gel pieces in 100 mM NH_4_HCO_3_ with 20 ng/μl trypsin/Lys-C (Promega) on ice for 30 min, after which excess liquid was removed and the gel pieces were incubated overnight at 37 °C. Trypsin/Lys-C was chosen for high fidelity and is expected to only partially cleave the RRKR motif to dicationic or tricationic peptides; tetra-cationic peptides are trypsin digested 100 times faster than dicationic and tricationic regions (56), and thus the full RRKR motif is not expected to remain after tryptic digestion. The gel pieces were washed with 5% v/v formic acid, and peptides were extracted by washing with 5% v/v formic acid and 50% v/v acetonitrile. The extracts were concentrated by lyophilization and stored at −20 °C until use in LC-MS/MS analysis.

### Liquid chromatography and MS

MS was performed at the RNA Epitranscriptomics & Proteomics Resource located at the Life Sciences Research Building, University at Albany, supported by the SUNY Research Foundation. LC-MS/MS was performed on an integrated micro LC-Orbitrap Velos system (Thermo Fisher Scientific), comprising a 3-pump Waters Cap LC microscale chromatography system (Waters Corp) with an autosampler, a stream-select module configured for precolumn plus analytical capillary column, and an Orbitrap Velos mass spectrometer fitted with an H-ESI probe operated under xcalibur 2.2 control.

Samples were resuspended in 0.1% v/v formic acid and 3% v/v acetonitrile. The peptides were separated on an ACE C18 capillary column (15 cm × 500 μm internal diameter) packed with ACE C18-300 particles (5 μm resin, Advanced Chromatography Technologies). The C18 column was connected in-line with the mass spectrometer. Peptides were eluted at a flow rate of 20 μl/min with a 40 min gradient of 5 to 80% acetonitrile in 0.1% formic acid into quartz emitters and analyzed by electrospray ionization MS, using a Orbitrap Velos mass spectrometer (Thermo Fisher Scientific) with an emitter voltage of 4.5 kV, a sheath gas flow of 10 and a capillary temperature of 275 °C. The Orbitrap was operated in a data-dependent acquisition mode in which multiple charged ions with abundance >6000 cps were selected for MS/MS fragmentation. Full scan mass spectra, 350 to 2000 *m/z*, with the resolution setting of 30,000 at 200 *m/z*, were detected in the Orbitrap analyzer after accumulation of 10^6^ ions. Peptides with multiple charges were selected automatically and fragmented in the collision cell through high-energy collision dissociation with 34% normalized collision energy at a resolution of 7500. A lock mass of diisooctyl phthalate, *m/z* 391.28428, was used for mass calibration. Data analysis was performed using pLink 2.3.9 (http://pfind.org/software/pLink/) software (57). Raw data from the LC-MS/MS experiments were deposited in the jPOSTrepo repository (58) under the accession codes JPST002156 and PXD042130.

### Cross-link MS analysis

Raw data from MS were processed using pLink 2.3.9 software (57) using the.RAW files and the following settings: flow type, high-energy collision dissociation; enzyme, trypsin, up to five missed sites; precursor mass tolerance, 20 ppm; fragment mass tolerance, 0.02 Da; peptide length, minimum three amino acids and maximum 60 amino acids per chain; peptide mass, minimum 300 and maximum 6000 Da; carbamidomethylation of cysteine, fixed; oxidation of methionine, variable; filter tolerance, 10 ppm; and false discovery rate <5% at the peptide-spectrum match level. Since only bacterially expressed or chemically synthesized and highly purified proteins were used in this study, the database searched consisted of the DID and DAD sequences and the masses used for cross-linked DSG, BS3, and BS(PEG)_5_ were 96.02113 Da, 138.06808 Da and 302.13656 Da, respectively. The results were annotated using pLabel (http://pfind.org/software/pLabel/index.html) (59), requiring a mass deviation <0.5 Da.

### Fluorescence titrations

Tryptophan fluorescence spectroscopy titration experiments were performed using a Fluorolog-3 Spectrofluorometer (Horiba). Excitation and emission wavelengths were 280 nm and 352 nm, respectively, with a slit width of 5 nm. Temperature was maintained at 20 °C and measurements were performed in triplicate. Protein samples were prepared in binding buffer, 10 mM 3-(N-morpholino)propanesulfonic acid, pH 7.5, 300 mM NaCl, and 0.01% NaN_3_, at 200 nM for WT DID and DID^E326G/E327A^ while DAD^M1199L^ concentrations ranged from 1 nM to 15 μM. The dissociation constant, K_D_, was calculated using the “one site – total, accounting for ligand depletion” regression model in the GraphPad Prism (https://www.graphpad.com/) version 9.0 software (GraphPad) using

$$\left[\text{DID}-{\text{DAD}}^{M1199L}\right]={\left[\text{DID}\right]}_{\text{total}}\;*\;{\left[{\text{DAD}}^{M1199L}\right]}_{\text{free}}/\left(K_{D}+{\left[{\text{DAD}}^{M1199L}\right]}_{\text{free}}\right)+\alpha {\left[{\text{DAD}}^{M1199L}\right]}_{\text{free}}$$

where [DID-DAD^M1199L^] is the amount of bound complex, K_D_ is the dissociation constant, and α is the nonspecific-binding coefficient (60). Through algebraic manipulations, the following quadratic is defined

$$\left(F-F_{o}\right)/F_{o}=\beta [\left(K_{D}+\left[{\text{DAD}}^{M1199L}\right]\right)\left(1+\alpha \right)+{\left[\text{DID}\right]}_{\text{total}}+\alpha \left[{\text{DAD}}^{M1199L}\right]+{\left[{\left(\left(K_{D}+\left[{\text{DAD}}^{M1199L}\right]\right)\left(1+\alpha \right)+{\left[\text{DID}\right]}_{\text{total}}+\alpha \left[{\text{DAD}}^{M1199L}\right]\right)}^{2}\;–\;4\left(1+\alpha \right)\left(\left[{\text{DAD}}^{M1199L}\right]\left(\alpha \left(K_{D}+\left[{\text{DAD}}^{M1199L}\right]\right)+{\left[\text{DID}\right]}_{\text{total}}\right)\right)\right]}^{1/2}]/\left[2\left(1+\alpha \right)\right]$$

where F is the fluorescence at a specific DAD^M1199L^ concentration, F_o_ is the fluorescence intensity of the blank, β is a scaling factor in the units of μM, [DAD^M1199L^] is the total ligand concentration and α = 0.00001.

### Stopped flow kinetics

Stopped flow fluorescence measurements were performed at 20 °C using a Stopped-Flow Module SFM-400 (Bio-Logic) equipped with an MPS-60 motor power supply, an ALX-250 arc lamp and a PMS-250 photo multiplier system. The tryptophan residue of DID was excited at 285 nm, and the fluorescence emission was measured at 305 nm with 700 V applied to the photo multiplier tube. Two hundred nanomolar (200 nM) DID, in binding buffer, was titrated from 1.5 μM to 20 μM with DAD^M1199L^. The measured voltage was plotted as a function of time and fit to a single exponential using a pseudo first-order kinetic model to estimate k_obs_, where k_obs_ = k_on_[DAD^M1199L^] + k_off_. Linear regression of k_obs_ *versus* [DAD^M1199L^] using Prism v9 software (GraphPad) yielded estimates for k_on_ (slope) and k_off_ (y-intercept), where k_on_ is the rate of complex formation and k_off_ is the rate of complex dissociation.

### NMR spectroscopy

All experiments were performed in 20 mM potassium phosphate, pH 7.5, 100 mM sodium chloride, 1 mM 4-(2-aminoethyl)benzenesulfonyl fluoride hydrochloride, 0.5 mM EDTA, 1 mM DTT, 15 mM HMPA, and 10% v/v D_2_O unless stated otherwise. NMR spectra were acquired using a 700 MHz Bruker Avance II or a 600 MHz Bruker Ascend spectrometer equipped with ultrasensitive cryoprobes. Experiments were performed with Watergate (24) water suppression, processed using TopSpin (https://www.bruker.com/en/products-and-solutions/mr/nmr-software/topspin.html) version 3.2 or 3.6, and assigned using Computer Aided Resonance Assignment (CARA, http://cara.nmr.ch) (25).

Three dimensional HNCA, HNCACB, CBCACONH and HNCO as well as 2D ^1^H-^15^N HSQC (38), spectra were recorded at 305 K to assign backbone residues of 250 μM [*U*-^15^N, ^13^C]-DID in the presence of a molar excess of WT DAD. To facilitate assignment of DID residues, 2D ^1^H-^15^N HSQC and 3D ^15^N NOESY spectra of DID bound to WT DAD with DID containing selectively ^15^N-labeled amino acids were recorded at 305 K or 298 K to facilitate DID resonance assignment. Selective labeling was accomplished for Ala, Asn, Met, Tyr, Cys, Arg, Gly, His, Ile, Leu, Lys, Phe, Thr, Trp, and Val.

Three dimensional HNCA and 2D ^1^H-^15^N HSQC spectra were recorded with 300 μM [*U*-^15^N,^13^C]-DID in the presence of 350 μM DAD^M1199L^ to facilitate transfer of H^N^, N, and CA assignments from DID-DAD spectra to DID-DAD^M1199L^ spectra. Side-chain proton assignments of [*U*-^15^N,^13^C]-DID bound to DAD^M1199L^ were obtained by recording H(CCCO)NH and 3D ^15^N-NOESY spectra (38). Backbone and side-chain assignments of free [*U*-^15^N,^13^C]-DAD^M1199L^ at 298 K were determined by recording (H)CCH-TOCSY, 3D CBCACONH, 2D ^1^H-^13^C HSQC, 2D ^1^H-^15^N HSQC, 3D HNCA, and 3D HNCOCA spectra (38). Backbone and side-chain assignments of [*U*-^15^N,^13^C]-DAD^M1199L^ bound to DID were determined by recording 3D HNCA, and 3D HNCOCA, (H)CCH-TOCSY and 3D HBHACONH at 305 K (38).

To determine temperature dependence of the transition between DID conformers A and B, 2D ^1^H-^15^N HSQC spectra were recorded with [*U*-^15^N]-DID bound to DAD^M1199L^ at 280, 283, 285, 288, 290, 292, 295, 298, 300, 303, 305, 308, and 310 K. The peak amplitudes of DID C-terminus residues G369, S373, Y374, and G378 were quantified. Equilibrium constants (K_eq_) were calculated for the transition between conformer A and B as the ratio of the cross-peak amplitudes of conformer A over conformer B. A linear fit of the van’t Hoff plot, ln(K_eq_) *versus* 1/T (K^−1^) was performed using GraphPad Prism 9.0 software. The linear regression analysis yielded thermodynamic parameters ΔG, ΔH, and ΔS for the conformer transition.

3D ^15^N-NOESY spectra were collected for [*U*-^15^N]-DID in the presence of excess unlabeled DAD^M1199L^ and for [*U*-^15^N]-DAD^M1199L^ in the presence of unlabeled DID. To facilitate the identification of intermolecular nOes, 3D ^15^N-NOESY spectra were collected for [*U*-^15^N]-DID in the presence of [*U*-^13^C]-DAD^M1199L^ in the presence and absence of a decoupling pulse on the ^13^C channel during ^1^H detection in the Bruker pulse program noesyhsqcf3gp193d.

Steady state {^1^H}-^15^N nOe spectra (61) were collected at 305 K using 450 μM [*U*-^15^N] DID with 650 μM unlabeled DAD^M1199L^ and 100 μM [*U*-^15^N]-DAD^M1199L^ with 200 μM unlabeled DID. Measurements of {^1^H}-^15^N nOes for free [*U*-^15^N]-DAD^M1199L^ were performed at 298 K to reduce exchange broadening. Interleaved spectra were recorded with and without proton saturation through a high-power 120° pulse applied every 5 ms within the 5 s recycle delay. Quantification of {^1^H}-^15^N nOes was performed using the equation {^1^H}-^15^N nOe = I_saturated_/I_unsat,_ where I_saturated_ is the peak amplitude from spectra collected with ^1^H saturation, and I_unsat_ corresponds to the condition without saturation. Error estimates of the {^1^H}-^15^N nOe, δ_nOe_, were determined from the noise levels in saturated, δ_saturated_, and unsaturated spectra, δ_unsat_, using the equation

$$\delta_{\text{nOe}}/\text{nOe}={\left[{\left(\delta_{\text{saturated}}/I_{\text{saturated}}\right)}^{2}+{\left(\delta_{\text{unsat}}/I_{\text{unsat}}\right)}^{2}\right]}^{1/2}$$

### NMR structure calculations

CYANA^26^ version 3.98.5 was used to calculate the 3D structure of DID in complex with DAD^M1199L^. The input consisted of a chemical shift list obtained from the resonance assignment, a sequence file of a single polypeptide chain in which the DID sequence was connected to DAD^M1199L^ *via* linker residues (LL5 in the standard CYANA library), an unassigned 3D ^15^N-NOESY peak list for DID, an unassigned 3D ^15^N-NOESY peak list for DAD^M1199L^, upper distance limits from cross-linking MS data (Table S3 footnote b), upper distance limits for manually assigned intramolecular and intermolecular nOes set at 5 Å, and distance restraints for helical regions identified in crystal structure PDB 2F31. Hydrogen bond N-O distances in acceptor-donor pairs were relaxed by 0.5 Å and N-C distances in helical regions were relaxed by 0.5 Å.

NOESY peaks were assigned automatically and converted into distance constraints using the standard CYANA macro with seven cycles of nOe assignment and simulated annealing in torsion angle space. The I-PINE NMR server (http://pine.nmrfam.wisc.edu) classified Pro 272 of DID as *cis* and all other DID prolines as *trans* based on the average Cβ chemical shifts (62), therefore Pro 272 was designated as cPro in the sequence file. The CISPROCHECK routine of CYANA 3.98.5 classified the isomerization state of Pro 1218 in DAD^M1199L^ as *trans* based on the average value and SD for the difference between the chemical shifts of Cβ and Cγ.^29^ Backbone Φ and Ψ dihedral angle constraints were determined using the TALOS-N webserver, (http://spin.niddk.nih.gov/bax/software/TALOS-N) (40). The experimental restraints used for the structure calculation are summarized in Tables S2 and S3.

CYANA-generated structures were subjected to minimization in explicit water using YASARA Structure version 20.12.24 (63). Distance restraints from manual and automatic assignment of nOes and cross-linking MS as well as the torsion angle list used in CYANA simulated annealing were used as input in the refinement procedure. In YASARA Structure version 20.12.24, refinement was performed using the Amber14 force field and the nmr_refinesolvent macro. Coordinates of the refined ensemble of 20 DID-DAD^M1199L^ structures were deposited in the Protein Data Bank with entry code 8FG1 (https://deposit-1.wwpdb.org/).

### Chemical shift index analysis

DID, DAD^M1199L^, and T-helix secondary structures were predicted using chemical shift index, CSI, analyses on the CSI 3.0 web server http://csi3.wishartlab.com. The consensus secondary structures were defined in terms of probabilities for a particular segment in DID/DAD^M1199L^ or T-helix to fold into α-helix, random coil, or β-sheet.

### Iso-surface map visualization

Iso-surface maps of the DID–DAD^M1199L^ and DID^E326G/E327A^–DAD^M1199L^ complexes were constructed using an optimized DID-DAD^M1199L^ structure from our refinements. Using the Adaptive Poisson-Boltzmann Solver (64) (https://server.poissonboltzmann.org/), the electronic surface properties of the proteins were calculated using the AMBER force field. Results were visualized using the Visual Molecular Dynamics (65) (https://www.ks.uiuc.edu/Research/vmd/) software with blue surfaces set to +10 eV and red surfaces set to −10 eV.

### Construction of plasmids for colocalization experiments

Preparation of plasmid constructs expressing human DIAPH1 and DIAPH1^ΔDAD^ with C-terminal monomeric enhanced YFP fluorescent tags, DIAPH1-YFP and DIAPH1^ΔDAD^-YFP, was described previously (10, 66). Constitutively active DIAPH1^ΔDAD^ was obtained by deleting the C-terminal DAD, which corresponds to amino acids 1194 to 1272 of DIAPH1. YFP fluorescent tags were excised from the *AgeI* and *NotI* sites of pCAG-YFP (Addgene) and *GFP* was inserted resulting in plasmids pDIAPH1-GFP and pDIAPH1^ΔDAD^-GFP. To obtain DIAPH1 constructs with N-terminal YFP tags, *YFP* was excised at the *AgeI* and *NotI* sites, and the gap was closed using a short linker duplex, 5′-CCGGTGTAATAGTGC-3′, 5′-GGCCGCGCACTATTACA-3′, containing two stop codons. *YFP* was amplified with two oligonucleotide primers 5′-TTAAGCGCTATGGTGAGCAAGGGCGAGGAG-3′ and 5′-TTACTCGAGACCGCCGCTACCGCCCTTGTACAGCTCGTCCATGCCGAG-3′, digested, and cloned into the *AfeI* and *XhoI* sites in upstream of the *DIAPH1* sequence resulting in plasmid pYFP-DIAPH1. Self-complementary oligonucleotides 5′-GCTCTCATCACACCAGCGGGCGCCCTTGACTTCCGAGTTCACA-3′ and 5′-TGTGAACTCGGAAGTCAAGGGCGCCCGCTGGTGTGATGAGAGC-3′ were used with the QuickChange Lightning site-directed Mutagenesis Kit (Agilent Technologies) to introduce mutations E326G/E327A into the *DIAPH1* sequence resulting in plasmids pYFP-DIAPH1^E326G/E327A^ and pDIAPH1^E326G/E327A^-GFP.

### HEK 293T cell culture, transfection, confocal microscopy

HEK 293T cells were grown in Dulbecco’s modified Eagle’s medium supplemented with 4 mM L-glutamine, 5.5 mM D-glucose, 1 mM sodium pyruvate, 10% fetal bovine serum (FBS), (HyClone), 100 units/ml penicillin, and 100 mg/ml of streptomycin. Fifteen millimeter (15 mm) diameter round glass coverslips (Ted Pella) were coated with Collagen I and seeded with 5 × 10^4^ cells each in the wells of 24-well plate. After incubating overnight at 37 °C, the Dulbecco’s modified Eagle’s medium was exchanged with Opti-minimal essential medium/5% FBS and the cells were transfected with plasmids using FuGENE 6 reagent according to the manufacturer’s recommendations. After 48 h of growth, cells were fixed by replacing the medium with 4% formaldehyde in PBS for 20 min at room temperature and washed three times with PBS. Cells were incubated with Alexa Fluor 568 Phalloidin (Thermo Fisher Scientific) solution in PBS, prepared in line with the manufacturer’s guidelines, for 45 min at 37 °C, washed three times with PBS, and mounted on slides using Fluoromount G (Electron Microscopy Sciences).

Images were acquired by using a Zeiss LSM 710 confocal imaging system (Carl Zeiss Microscopy) equipped with an X63, 1.4 NA oil immersion objective. GFP was excited with a 488 nm argon laser line at 1% power and Alexa Fluor 568 with a 561 nm line at 0.8% power. Image stacks were recorded at 290 nm steps along the *z*-axis in two-channel mode, using a 488/561 nm beam splitter with a 492 to 550 nm emission window for the GFP channel and a 592 to 676 nm window for the Alexa Fluor 568 channel. A total of eight Z-stacks were acquired for DIAPH1^ΔDAD^-GFP/F-Actin cells, and nine each for DIAPH1^E326G/E327A^-GFP/F-Actin and DIAPH1-GFP/F-Actin cells.

3D images were processed using FluoRender 2.27 software (67) (https://www.sci.utah.edu/software/fluorender.html). ImageJ plugin JACoP 2.0 (68) (https://imagej.nih.gov/ij/plugins/track/jacop2.html) was used to assess the degree of colocalization of F-Actin with DIAPH1 or its mutants. Z-stacks were processed at default settings in 3D mode, and Manders coefficients M1 and M2, which measures the amount of colocalization for each image were recorded. Colocalization data was processed with Prism 9.0.2 (https://www.graphpad.com/) software (GraphPad). Column heights reported on the graph represent mean values, error bars represent the SD. The statistical significance of multiple comparisons was estimated by using the one-way ANOVA subroutine and Fisher’s least significant difference test with a single pooled variance displayed in GraphPad style, where the absence of bars represents *p* > 0.05, ∗ is *p* ≤ 0.05, ∗∗ is *p* ≤ 0.01, and ∗∗∗ is *p* ≤ 0.001.

### Human primary aortic vascular SMC migration

Human primary aortic vascular SMCs (ATCC PCS-100-012) were purchased from American Type Tissue Culture Collection. Cells were propagated in Vascular Cell Basal Medium (ATCC PCS-100-030) supplemented with the Vascular Smooth Muscle Cell Growth Kit (ATCC PCS-100-042) containing recombinant human, rh-FGF-b, rh-insulin, rh-EGF, ascorbic acid, L-glutamine, FBS, and 0.1% penicillin/streptomycin in a humidified atmosphere of 5% CO_2_ at 37 °C.

Lipofectamine LTX reagent (Thermo Fisher Scientific) was used to perform transfection according to manufacturer’s protocol. The protocol was optimized for 2.5 μg/ml of plasmid DNA in reduced serum Opti-minimal essential medium for efficient transfection. Human primary aortic SMCs were assessed for migration in response to 10 μg/ml of RAGE ligand, CML-HSA (8) with a wounding assay as previously described (69). Briefly, cells were grown to confluence in 24-well plates and serum-starved overnight. The following morning, the monolayer was wounded using a p10 pipette tip and fresh medium containing RAGE ligand was added for 4 h. Cells were maintained at 37 °C and 5% CO_2_. Images were taken at 4 h intervals. Each image was measured, and an ingrowth area of effective migrating cells was calculated. The data was subjected to a Grubbs test with α = 0.1, and the resulting data was processed with Prism v 9.0.2 software (GraphPad). Column heights reported on the graph represent mean values, error bars represent the SD. The statistical significance of multiple comparisons was estimated by using the one-way ANOVA subroutine.

## Data availability

• Expression plasmids generated in this study are available upon request from the corresponding author with a completed materials transfer agreement.
• Chemical shift assignments were deposited into the Biological Magnetic Resonance Bank with accession code: 31064.
• Distance restraints and atomic coordinates for reported structures are deposited into the Protein Data Bank with accession code: 8FG1.
• MS data was deposited to https://repository.jpostdb.org/under acquisition codes JPST002156 and PXD042130.

## Supporting information

This article contains supporting information (57, 60, 70, 71).

## Conflict of interest

The authors declare that they have no conflicts of interest with the content of this article.
