# Supplementary material for: Disruption of the productive encounter complex results in dysregulation of DIAPH1 activity
Source: J Biol Chem. 2023 Oct 12;299(11):105342. doi: 10.1016/j.jbc.2023.105342 (PMC10656230; doi:10.1016/j.jbc.2023.105342)
Supplement: Supporting Figures S1–S6 and Tables S1–S5 [file mmc1.pdf]

Supplementary Materials.

Disruption of the Productive Encounter Complex Results in Dysregulation of DIAPH1 Activity

Gregory G. Theophall[1$], Lisa M. S. Ramirez[1$+], Aaron Premo[1], Sergey Reverdatto[1], Michaele B. Manigrasso[2], Gautham Yepuri[2], David S. Burz[1], Ravichandran Ramasamy[2], Ann Marie Schmidt[2], Alexander Shekhtman[1*]

[1]Department of Chemistry, State University of New York at Albany, Albany, NY 12222

[2]Diabetes Research Program, Department of Medicine, New York University Grossman School of Medicine, New York, NY 10016

[*]Corresponding author, email: ashekhtman@albany.edu

[$]Contributed equally to the paper

[+]Present address is: German Center for Neurodegenerative Diseases (DZNE), Goettingen, Germany, 37075

Supplementary Information

Figure S1. Stoichiometry and affinity of the DID-DAD^{M1199L} interaction.

Figure S2. DAD mutation M1199L results in a minor structural perturbation of the DID-DAD complex.

Figure S3. DAD^{M1199L}-DID cross-links observed by MS.

Figure S4. Carboxy terminus of DAD^{M1199L} exhibits variable orientations.

Figure S5. T-helix forms a transient helix and does not compete with DAD^{M1199L} for binding to DID.

Figure S6: Mutations E326G/E327A do not alter DID tertiary structure in solution.

Table S1. Thermodynamic parameters resolved for the DID C-terminal B (unfolded) to A (folded) conformer transition.

Table S2. Structural statistics of the ensemble of 20 DID-DAD^{M1199L} conformers.

Table S3. Inter- and intramolecular cross-links between DID and DAD^{M1199L} used in structure calculations.

Table S4. Inter- and intramolecular cross-links between DID and DAD^{M1199L} *not* used in structure calculations.

Table S5. Chemical shift assignments of T-helix.

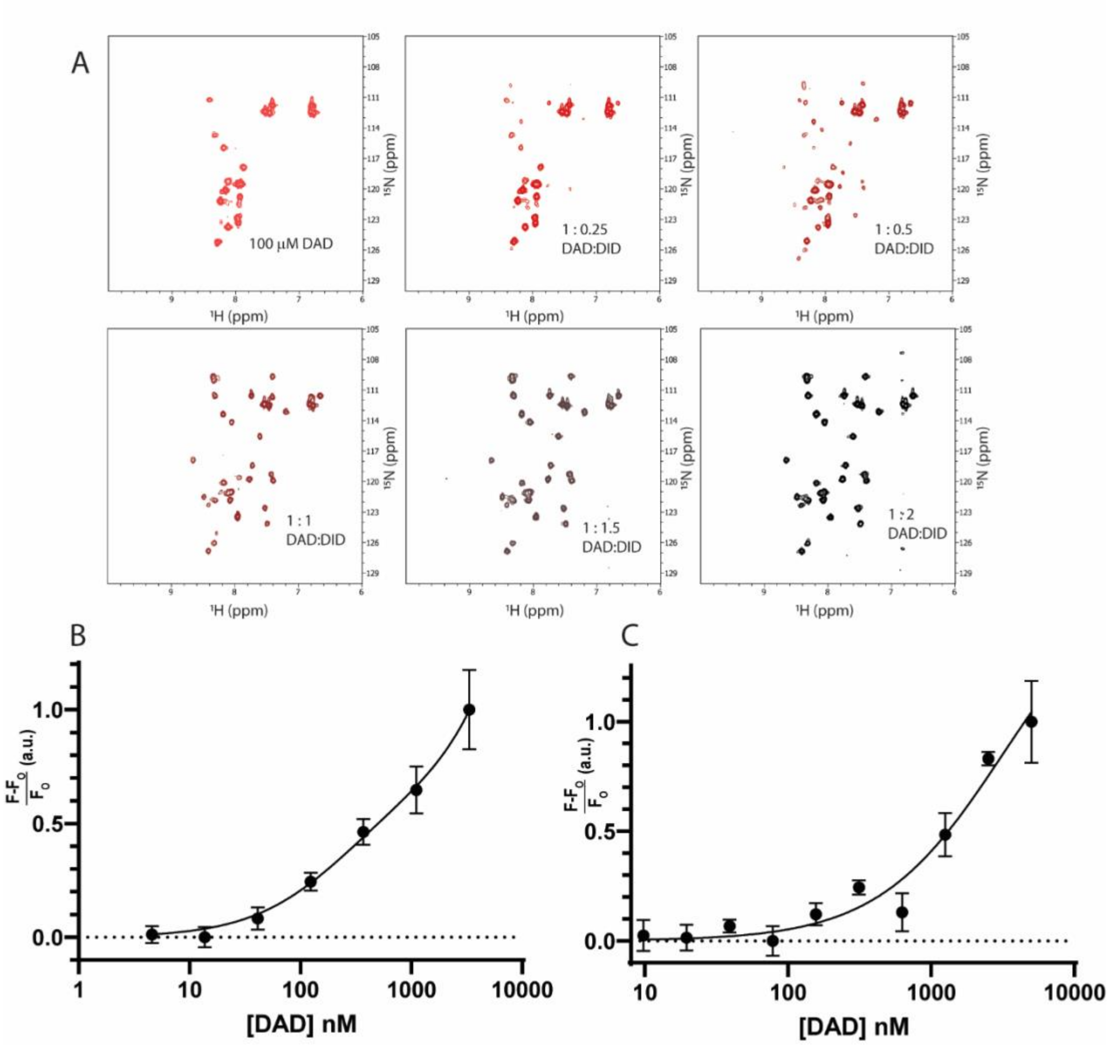

Figure S1. **Stoichiometry and affinity of the DID-DAD^{M1199L} interaction**. A) ^{1}H-^{15}N HSQC spectrum of 100 μM [*U*-^{15}N]-DAD^{M1199L} with increasing amounts of unlabeled DID. Perturbations in chemical shifts and intensities reach saturation at a 1:1 mole ratio. B-C) Fluorescence titrations of 200 nM wild type DID (B) and 200 nM DID$^{E326G/E327A}$ (C) with DAD^{M1199L}. Fitting the data with a total binding, accounting for ligand depletion,[1] binding curve yielded dissociation constants, K_D, for the interaction.

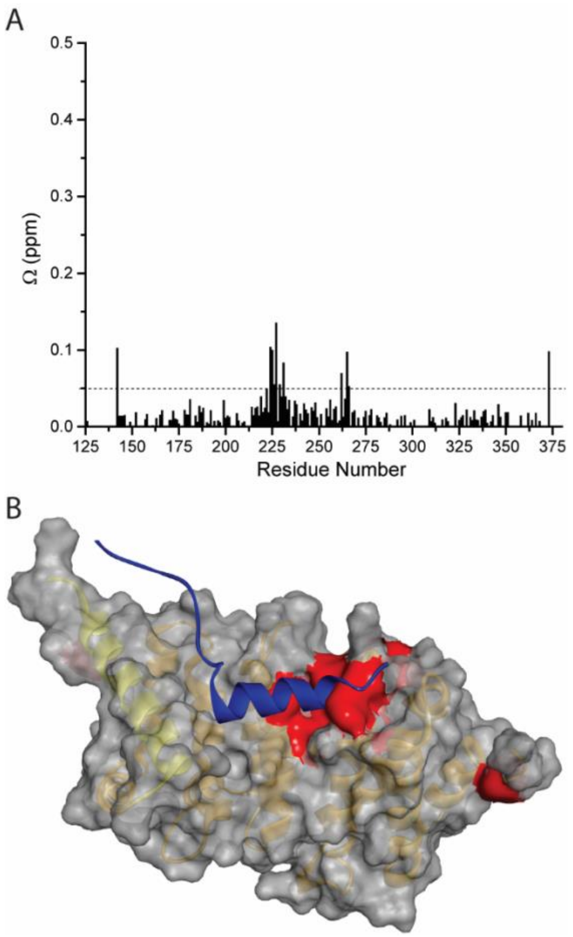

Figure S2. DAD mutation M1199L results in a minor structural perturbation of the DID-DAD complex. A) Chemical shift changes, Ω, between [*U*-^{15}N]-DID-DAD and [*U*-^{15}N]-DID-DAD^{M1199L} complexes. B) Chemical shift changes mapped onto a semi-transparent space filling model of DID (grey) showing Ω >0.05 in red. The core structure of DID is tinted brown and the interhelix domain is yellow. Chemical shift changes are calculated as $\Omega = \sqrt{((\Delta H)^2 + (\Delta N/2)^2)}$, where ΔH and ΔN are changes in chemical shift in the hydrogen and nitrogen dimension, respectively. The DAD$^{\mathbf{M1199L}}$ peptide is shown as a blue ribbon. The cutoff value for significant changes, Ω = 0.05, defines the largest 3% of the Ω values.

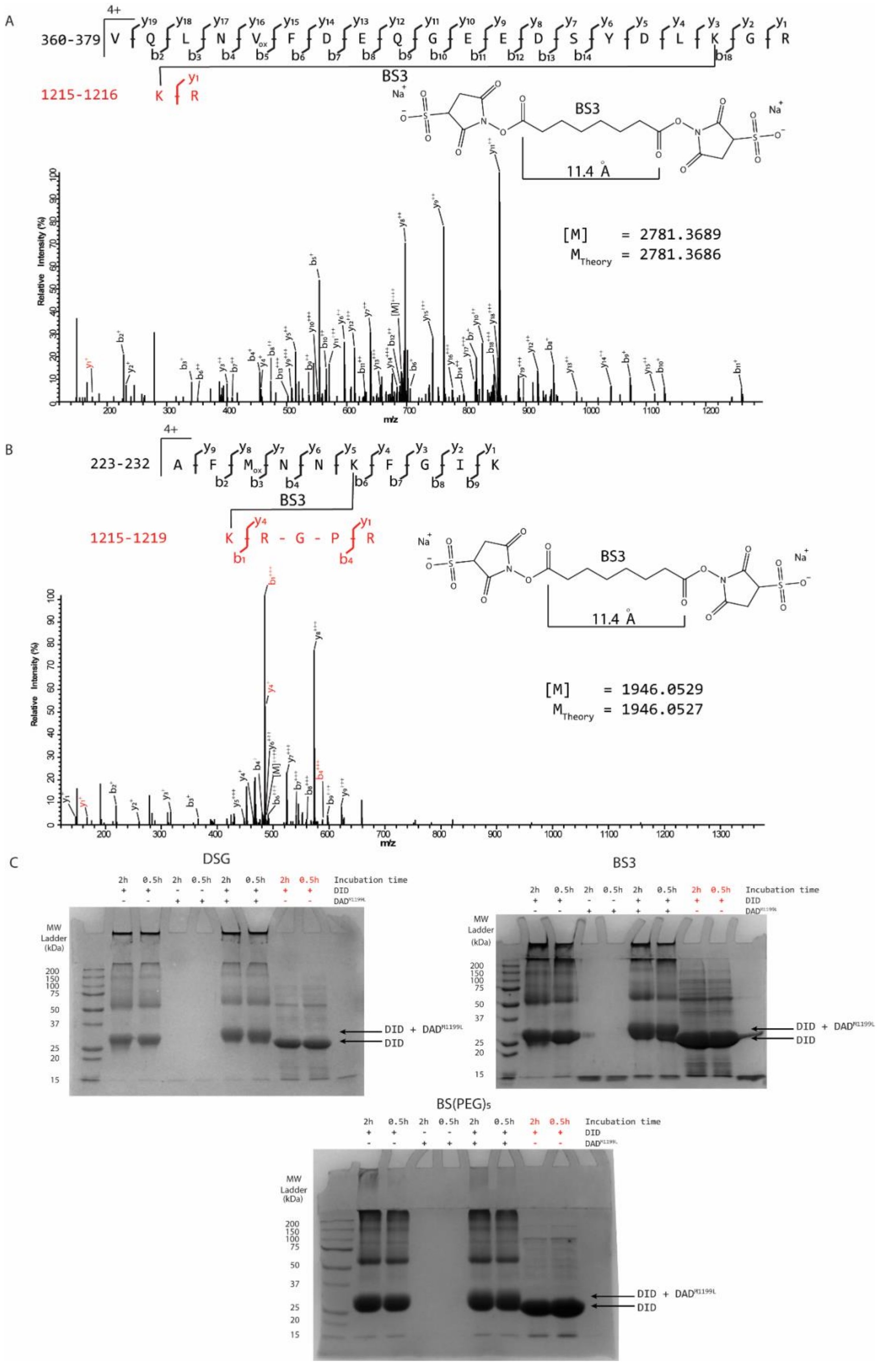
A
360-379
V Q L N V F D E Q G E E D S Y D L K G R
1215-1216
K R
BS3
Na
11.4 Å
Relative Intensity (%)
m/z
[M] = 2781.3689
M Theory = 2781.3686
B
223-232
A F M N N K F G I K
1215-1219
K R - G - P R
BS3
[M] = 1946.0529
M Theory = 1946.0527
C
DSG
2h 0.5h 2h 0.5h 2h 0.5h 2h 0.5h Incubation time
DID
MW Ladder (kDa)
200
150
100
75
50
37
25
20
15
DID + DAD M1199L
DID
BS3
BS(PEG)5

Figure S3. Cross-links of DAD^{M1199L} with DID observe by MS. A). High-energy collision MS spectrum obtained for the BS3 cross-linked product between K377 of DID and K1215 of DAD^{M1199L} at 695.327 m/z. For clarity, only the molecular ion, and the y and b peptidic fragmentations are labeled. The cross-linked peptides are shown in black and red along with the cross-linker BS3. The experimental mass of 2781.3689 Da is in good agreement with the theoretical mass of 2781.3686 Da calculated from putative elemental composition. Unassigned peaks are from water or ammonia loss, and/or non-peptidic bond fragmentation. The charge of the peptide fragment is shown on the top left corner of the sequence. B). High-energy collision MS spectrum obtained for the BS3cross-linked product between K228 of DID and K1215 of DAD^{M1199L} at 486.63 m/z. For clarity, only the molecular ion, and the y and b peptidic fragmentations are labeled. The cross-linked peptides are shown in black and red along with the cross-linker BS3. The experimental mass of 1946.0529 Da is in good agreement with the theoretical mass of 1946.0527 Da calculated from putative elemental composition. Unassigned peaks are from water or ammonia loss, and/or non-peptidic bond fragmentation. The charge of the peptide fragment is shown on the top left corner of the sequence. C). 8% SDS-PAGE gel of DID and DAD^{M1199L} with cross-linkers DSG, BS3, and BS(PEG)$_5$. The + and – symbols indicate the presence or absence of the protein, respectively, and the red labels denote no cross-linker was added.

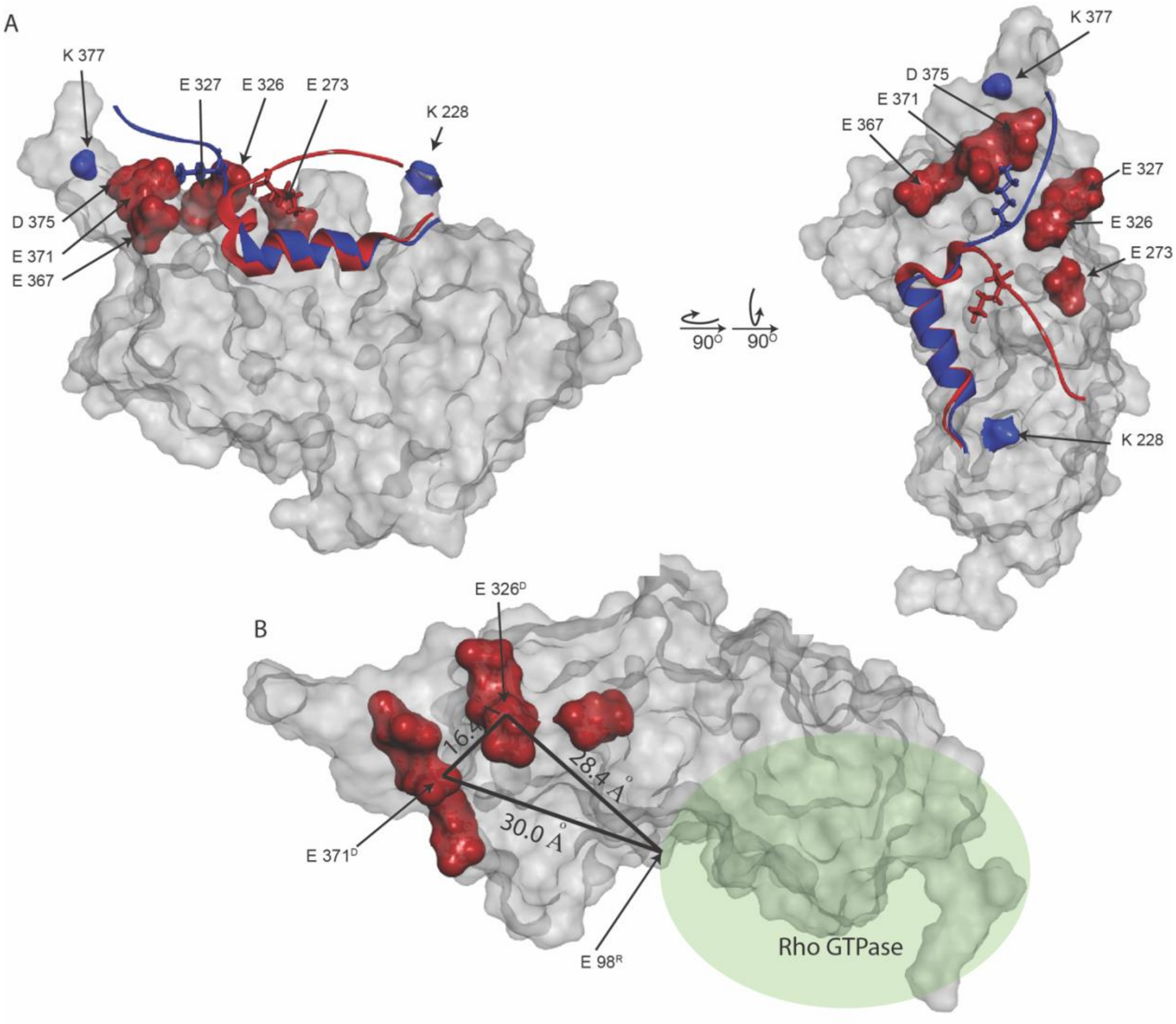

Figure S4. The carboxy terminus of DAD^{M1199L} exhibits variable orientations. A) YASARA-refined model of the DID molecular surface showing selected lysine residues involved in DID-DAD^{M1199L} cross-links (blue surface) and acidic residues which interact with the RRKR motif (red surface). The orientation of the DAD^{M1199L} carboxy terminus (ribbon structure) varies depending on the distance constraints used in refinement: cross-link between DID K228 and DAD^{M1199L} K1215 (red) and DID K377 and DAD^{M1199L} K1215 (blue). B) The RRKR binding site is located ~ 30 Å away from the Rho GTPase binding site (PDB: 1Z2C). Triangulation of the acidic patch of DID (red) with respect to the Rho GTPase binding site (green oval). Residues used are labeled with a D superscript for the RRKR and R for the Rho GTPase binding sites.

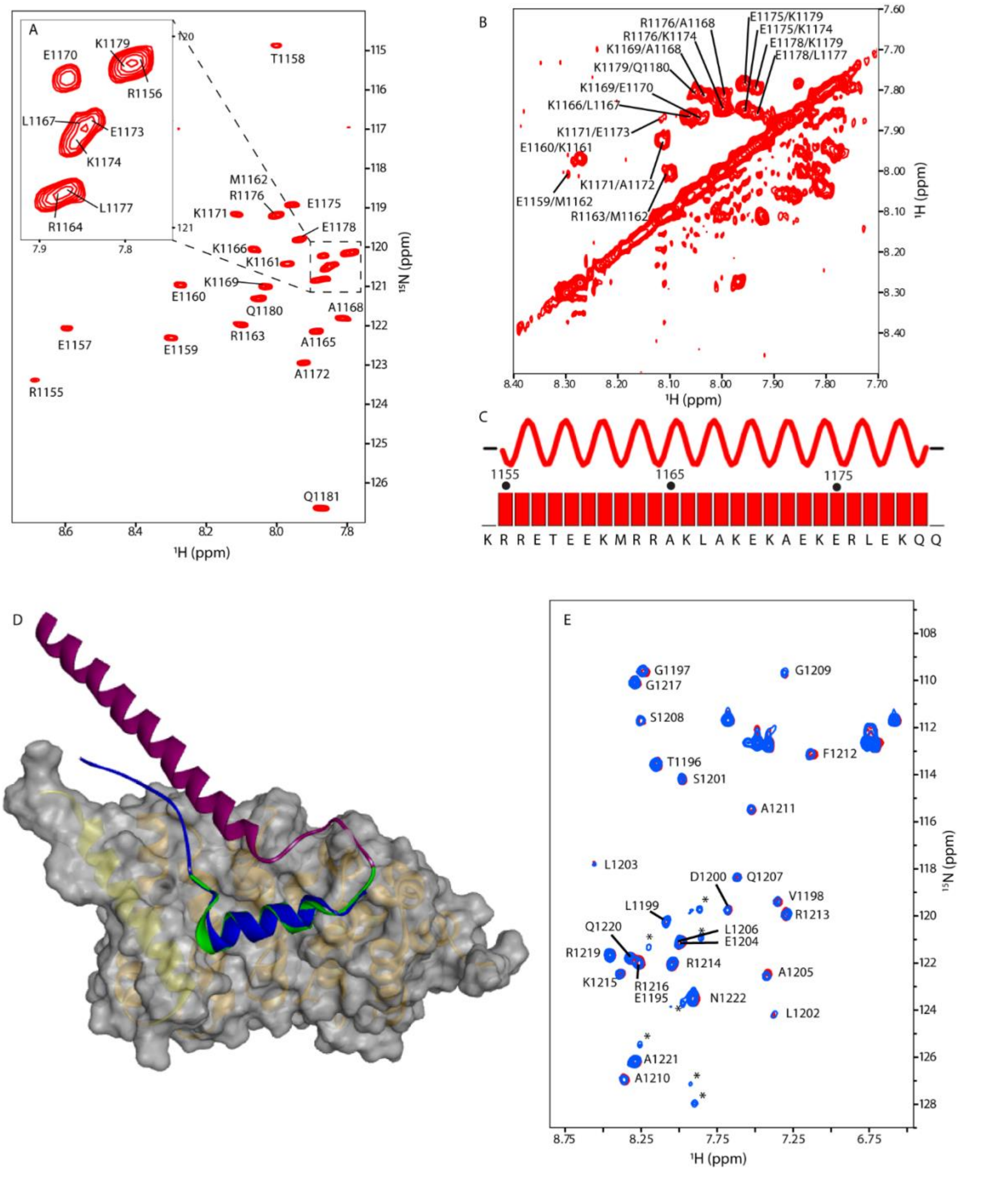

Figure S5. T-helix forms a transient helix and does not compete with DAD^{M1199L} for binding to DID. A) ^{1}H-^{15}N HSQC spectrum of the T-helix portion of the FH2 domain in DIAPH1 shows a wide dispersion of amide proton resonances indicating the presence of secondary structure. B). ^{1}H-^{1}H NOESY spectrum of the T-helix with amide cross peaks labeled. C). Chemical Shift Index[2] 3.0 analysis predicts an α-helical structure for the T-helix (**Table S5**). D). Superposition of a semi-transparent space filling model of DIAPH1 DID and DAD^{M1199L} (blue ribbon) with the T-helix (red ribbon) and DAD (green ribbon) of mDia1 (PDB 3OBV) shows that the T-helix (red ribbon)

and DAD (blue ribbon) binding sites overlap. The core structure of DID is tinted brown and the interhelix domain is yellow. The orientation is the same as in Figure 3D. E). ^{1}H-^{15}N HSQC spectrum of [*U*-^{15}N]-DAD^{M1199L} bound to DID in the absence of T-helix (red), and with a 3-fold molar excess of T-helix (blue). No visible changes in the NMR peaks suggest that the T-helix does not compete with DAD for DID in solution. Peaks from DAD^{M1199L} degradation are marked with asterisks.

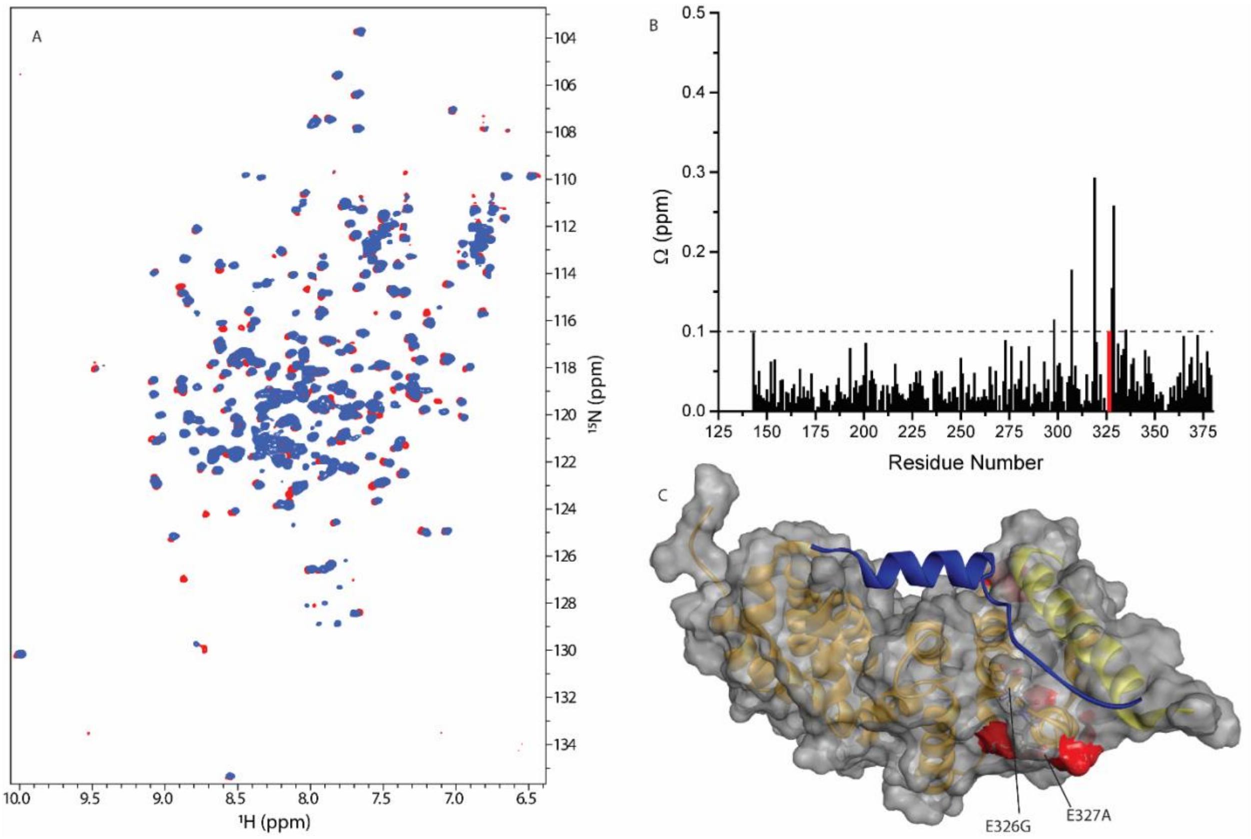

Figure S6: Mutations E326G/E327A do not alter DID tertiary structure in solution. A). Superimposed ^{1}H-^{15}N HSQC spectra of [*U*-^{15}N]-DID (red) and [*U*-^{15}N]-DID$^{E326G/E327A}$ (blue) in the presence of a molar excess of unlabeled DAD^{M1199L}. B). Chemical shift changes, Ω, of the [*U*- ^{15}N]-DID$^{E326G/E327A}$-DAD^{M1199L} complex cross peaks relative to [*U*-^{15}N]-DID-DAD^{M1199L}. The point mutations E326G and E327A are shown in red. C). Semi-transparent space filling model of DID showing $\Omega > 0.1$ ppm in red. The ribbon structure of DID is tinted brown and the interhelix domain is yellow. The DAD$^{\mathbf{M1199L}}$ peptide is shown as a blue ribbon. Orientation is identical to **Fig. 3D**. Ω was calculated as $\sqrt{((\Delta H)^2 + (\Delta N/2)^2)}$, where ΔH and ΔN are ppm changes in chemical shifts in the hydrogen and nitrogen dimensions, respectively. The cutoff value for significant changes, $\Omega = 0.1$, defines the largest 3% of the Ω values.

Table S1. Thermodynamic parameters resolved for the DID C-terminal B (unfolded) to A (folded) conformer transition.

	ΔH (kJ/mol)	ΔS (kJ/mol-K)	TΔS (kJ/mol)	ΔG (kJ/mol)
G369	45.6 ± 3.0	0.14 ± 0.010	42.2 ± 3.2	3.4 ± 4.4
S373	55.0 ± 3.1	0.17 ± 0.010	53.2 ± 3.2	1.8 ± 4.5
Y374	49.8 ± 2.9	0.15 ± 0.010	47.1 ± 3.0	2.7 ± 4.2
G378	54.0 ± 1.6	0.18 ± 0.005	54.3 ± 1.6	-0.3 ± 2.3

Table S2. Structural statistics of the ensemble of 20 DID-DAD^{M1199L} conformers.

Parameter	Value		
Distance restraints from nOes	nOes assigned by CYANA[a]	Manually assigned nOes[b]	Total nOes
nOe upper distance limits			
Intra-residue (*i*=*j*) in DID	314	16	330
Short-range |(*i*-*j*)|= 1 in DID	410	39	449
Medium-range |(*i*-*j*)| ≤5 in DID	80	15	95
Long-range |(*i*-*j*)| >5 in DID	13	14	27
DID intramolecular nOes			901
Intra-residue (*i*=*j*) in DAD^{M1199L}	36	0	36
Short-range |(*i*-*j*)|= 1 in DAD^{M1199L}	43	1	44
Medium-range |(*i*-*j*)| ≤5 in DAD^{M1199L}	13	0	13
Long-range |(*i*-*j*)| >5 in DAD^{M1199L}	1	0	1
DAD^{M1199L} intramolecular nOes			94
DID-DAD^{M1199L} intermolecular nOes	10	3	13
Violations[c]			
nOe[c]	38 violations (0.4 ±0.3 Å)		
torsion angles[c]	9 violations (16 ±15°)		
Ramachandran statistics[c]	87.1% in most favored regions<br>12.3% in additionally allowed regions<br>0.5% in generously allowed regions<br>0.1% in disallowed regions		
All-atom clash score[d]	2		
RMSD for well-defined (core) regions[e]	0.33 Å		

a) nOes automatically assigned by CYANA version 3.98.5 (https://www.cyana.org/) following 7 cycles of automated nOe assignment, nOes are counted without pseudo-atom expansion.
b) nOes manually assigned without pseudo-atom expansion.
c) Calculated using CYANA 3.98.5 prior to refinement in solvent corresponding to violations in 6 or more structures of the ensemble above the cut-offs: 0.2 Å for nOe upper limits, 5° for torsion angles.
d) The all-atom clash score represents the number of atom clashes per 1000 atoms.
The DID residue ranges are 145-200, 209-268, 275-373 and DAD^{M1199L} residues are 1197-1212.

d-e) Taken from the validation report of the deposited coordinates (PDB code 8FG1) in the Protein Data Bank (https://deposit-1.wwpdb.org/).

Table S3. **Inter- and intramolecular cross-links between DID and DAD^{M1199L} used in structure calculations.**

Cross-linker reagent	Disuccinimidyl glutarate,<br>DSG	Bis(sulfosuccinimidyl) suberate,<br>BS3[a]	Bis(sulfosuccinimidyl) suberate,<br>BS3[a,b]
Spacer length	7.7 Å	11.4 Å	11.4 Å
Specificity	Homobifunctional<br>Primary amines	Homobifunctional<br>Primary amines	Homobifunctional<br>Primary amines
Sequences of cross-linked peptides	^{1194}DETGVLDSLLEALQSGAAFR^{1214}<br>^{223}AFMNN**K**FGIK^{232}<br>4 spectra – charge +3[d]	^{223}AFMNN**K**FGIK^{232}<br>^{1215}**K**RGPR^{1219}<br>(oxidized M234)<br>2 spectra – charge +2[d]	^{360}VQLNVFDWQGEEDSYDL**K**<br>GR^{380}<br>^{1215}KR^{1216}<br>1 spectrum – charge +4[d]
Cross-linked residues	DAD^{M1199L} N-terminus – DID K228<br>INTERMOLECULAR	DID K228 – DAD^{M1199L} K1215<br>INTERMOLECULAR	DID K377 – DAD^{M1199L} K1215<br>INTERMOLECULAR
$M_{theoretical}$	3356.6830 Da	1946.0527 Da	2781.3686 Da
$[M]_{experimental}$	3356.6906 Da	1946.0529 Da	2781.3689 Da
Error	2.3 ppm	0.10 ppm	-0.11 ppm
Distance violation	corresponds to 7.8 Å distance in mDia (PDB 2F31), 0.1 Å above expected spacer length	corresponds to 30.5 Å distance in mDia (PDB 3O4X), 19.1 Å above expected spacer length	corresponds to 12.1 Å distance in mDia (PDB 3O4X), 0.7 Å above expected spacer length
Score[c]	0.0258	0.0130	0.102

Cross-linker reagent	Disuccinimidyl glutarate,<br>DSG	bis(succinimidyl)penta(ethylene glycol), BS(PEG)$_5$
Spacer length	7.7 Å	21.7 Å
Specificity	Homobifunctional<br>Primary amines	Homobifunctional<br>Primary amines
Sequences of cross-linked peptides	^{198}LHDE**K**EETAGSYDSR^{212}<br>^{213}N**K**HEIIR^{219}<br>3 spectra – charge +4[d]<br>3 spectra – charge +5[d]	^{295}FQPLLDGL**K**SGTTIALK^{311}<br>^{198}LHDE**K**EETAGSYDSR^{212}<br>1 spectrum – charge +5[d]
Cross-linked residues	DID K202 – DID K214<br>INTRAMOLECULAR	DID K303 – DID K202<br>INTRAMOLECULAR
$M_{theoretical}$	2741.3124 Da	3839.9428 Da
$[M]_{experimental}$	2741.3117 Da	3839.9435 Da
Error	-0.26 ppm	-0.18 ppm
Distance violation	corresponds to 7.8 Å distance in mDia (PDB 2F31), 0.1 Å above expected spacer length (K214 is Q205 in mDia)	corresponds to 16.7 Å distance in mDia (PDB 2F31), 5 Å below expected spacer length
Score[c]	0.00398	0.104

a.) This set of cross-links cannot exist concurrently and each cross-link was employed independently during structural calculations. Cross-link with footnote b was used in the PDB submission, see materials and methods.
b.) The assignment of KR to be part of the RRKR motif and not part of DID ^{196}KR is based on the distance constraint set by the cross-linker BS3.
c.) The score value is from pLink2[3] 2.3.9 output file. The range of the score values fall within the expected 1 to 10^{-3} values from a pLink analysis of a cross-linked synthetic peptide library.[4]
d.) MS data were deposited to https://repository.jpostdb.org/ under acquisition codes JPST002156 and PXD042130.

Table S4. **Inter- and intramolecular cross-links between DID and DAD^{M1199L} *not* used in structure calculations.**

Cross-linker reagent	Bis(sulfosuccinimidyl) suberate, BS3[a]	Bis(sulfosuccinimidyl) suberate, BS3[a]	bis(succinimidyl)penta(ethylene glycol), BS(PEG)$_5$[b]
Spacer length	11.4 Å	11.4 Å	21.7 Å
Specificity	Homobifunctional<br>Primary amines	Homobifunctional<br>Primary amines	Homobifunctional<br>Primary amines
Sequences of cross-linked peptides	^{223}AFMNN**K**FGIK^{232}<br>^{1215}**K**R^{1216}<br>2 spectra – charge +2[d]<br>2 spectra – charge +3[d]	^{1194}DETGVLDSLLEALQSGAA FR^{1214}<br>^{223}AFMNN**K**FGIK^{232}<br>2 spectra – charge +3[d]<br>2 spectra – charge +4[d]	^{223}AFMNN**K**FGIK^{232}<br>^{1215}**K**R^{1216}<br>1 spectrum – charge +2[d]
Cross-linked residues	DID K228–DAD^{M1199L} K1215 INTERMOLECULAR	DAD^{M1199L} N-terminus – DID K228 INTERMOLECULAR	DID K228–DAD^{M1199L} K1215 INTERMOLECULAR
$M_{theoretical}$	1609.8882 Da	3398.7299 Da	1773.9567 Da
$[M]_{experimental}$	1609.8870 Da	3398.7319 Da	1773.9594 Da
Error	-0.75 ppm	0.59 ppm	1.53 ppm
Distance violation	corresponds to 30.5 Å distance in mDia (PDB 3O4X), 19.1 Å above expected spacer length	corresponds to 7.8 Å distance in mDia (PDB 2F31), 3.6 Å above expected spacer length	corresponds to 30.5 Å distance in mDia (PDB 3O4X), 8.9 Å above expected spacer length
Score[c]	0.00708	0.000651	0.258

Cross-linker reagent	bis(succinimidyl)penta(ethylene glycol), BS(PEG)$_5$[b]	bis(succinimidyl)penta(ethylene glycol), BS(PEG)$_5$[b]	Bis(sulfosuccinimidyl) suberate, BS3[a]
Spacer length	21.7 Å	21.7 Å	11.4 Å
Specificity	Homobifunctional<br>Primary amines	Homobifunctional<br>Primary amines	Homobifunctional<br>Primary amines

Sequences of cross-linked peptides	^{223}AFMNN**K**FGIK^{232}<br><br>^{1215}**K**R^{1216}<br>(oxidized M234)<br>1 spectrum – charge +2[d]<br>1 spectrum – charge +3[d]	^{223}AFMNN**K**FGIK^{232}<br><br>^{1214}R**K**R^{1216}<br>(oxidized M234)<br>1 spectra – charge +4[d]	^{198}LHDE**K**EETAGSYDSR^{212}<br>^{213}N**K**HEIIR^{219}<br>1 spectra – charge +4[d]<br>1 spectra – charge + 5[d]<br>2 spectra – charge +6[d]
Cross-linked residues	DID K228–DAD^{M1199L} K1215<br>INTERMOLECULAR	DID K228–DAD^{M1199L} K1215<br>INTERMOLECULAR	DID K202 – DID K214<br>INTRAMOLECULAR
$M_{theoretical}$	1789.9516 Da	1946.0527 Da	2783.3593 Da
$[M]_{experimental}$	1789.9529 Da	1946.0529 Da	2783.3517 Da
Error	0.72 ppm	0.11 ppm	-2.74 ppm
Distance violation	corresponds to 30.5 Å distance in mDia (PDB 3O4X), 8.9 Å above expected spacer length	corresponds to 30.5 Å distance in mDia (PDB 3O4X), 8.9 Å above expected spacer length	corresponds to 7.8 Å distance in mDia (PDB 2F31), 3.6 Å above expected spacer length (K214 is Q205 in mDia)
Score[c]	0.177	0.182	0.00398

a.) These cross-links were not used because of redundancy.
b.) These cross-links were not used due to low statistical properties despite low mass errors.
c.) The score value is from pLink2[3] 2.3.9 output file. The range of the score values fall within the expected 1 to 10^{-3} values from a pLink analysis of a cross-linked synthetic peptide library.[4]
d.) MS data were deposited to https://repository.jpostdb.org/ under acquisition codes JPST002156 and PXD042130.

Table S5. Chemical shift assignments of T-helix.

Residue	H-N (ppm)	H-Alpha (ppm)	H-Beta (ppm)	C-Alpha (ppm)
K1153	N/A	N/A	N/A	N/A
R1154	8.68	4.12	1.66/1.68	53.58
R1155	7.79	3.98	1.78/1.71	55.02
E1156	8.59	4.12	1.90/1.84	54.07
T1157	8.00	3.96	4.18	60.97
E1158	8.30	3.95	1.92	55.92
E1159	8.27	3.988	1.84/1.92	55.78
K1160	7.97	3.843	N/A	56.46
M1161	8.01	4.11	2.55/2.39	53.09
R1162	8.10	3.93	1.76	56.06
R1163	7.88	3.973	N/A	56.02
A1164	7.89	4.02	1.32	51.59
K1165	8.07	3.93	N/A	55.79
L1166	7.86	4.05	N/A	53.30
A1167	7.82	4.05	1.32	51.58
K1168	8.03	3.94	N/A	55.82
E1169	7.87	4.00	N/A	55.93
K1170	8.12	3.87	1.74	56.11
A1171	7.93	4.07	1.33	51.33
E1172	7.88	3.94	1.75	56.12
K1173	7.85	3.93	N/A	55.72
E1174	7.96	3.94	2.07/2.12	55.26
R1175	8.00	4.01	N/A	54.25
L1176	7.86	4.0	N/A	54.37
E1177	7.94	4.02	1.87/2.13	55.34
K1178	7.80	4.12	N/A	55.01
Q1179	8.05	4.14	1.87/1.99	53.93
Q1180	7.88	3.98	1.97	54.51

References.

1 Swillens, S. Interpretation of binding curves obtained with high receptor concentrations: practical aid for computer analysis. *Mol Pharmacol* **47**, 1197-1203 (1995).

2 Wishart, D. S. & Sykes, B. D. The 13C chemical-shift index: a simple method for the identification of protein secondary structure using 13C chemical-shift data. *J Biomol NMR* **4**, 171-180 (1994).

3 Chen, Z. L. *et al.* A high-speed search engine pLink 2 with systematic evaluation for proteome-scale identification of cross-linked peptides. *Nat Commun* **10** (2019). https://doi.org:10.1038/s41467-019-11337-z

4 Beveridge, R., Stadlmann, J., Penninger, J. M. & Mechtler, K. A synthetic peptide library for benchmarking crosslinking-mass spectrometry search engines for proteins and protein complexes. *Nat Commun* **11**, 742 (2020). https://doi.org:10.1038/s41467-020-14608-2
